# Psychrophilic phage VSW-3 RNA polymerase reduces both terminal and full-length dsRNA byproducts in *in vitro* transcription

**DOI:** 10.1080/15476286.2022.2139113

**Published:** 2022-10-26

**Authors:** Heng Xia, Bingbing Yu, Yixin Jiang, Rui Cheng, Xueling Lu, Hui Wu, Bin Zhu

**Affiliations:** a Key Laboratory of Molecular Biophysics, the Ministry of Education, College of Life Science and Technology, Huazhong University of Science and Technology, Wuhan, China; b Shenzhen Huazhong University of Science and Technology Research Institute, Shenzhen, China

**Keywords:** dsRNA, sgRNA, mRNA medicine, T7 RNA polymerase, in vitro transcription

## Abstract

RNA research and applications are underpinned by *in vitro* transcription (IVT), but RNA impurities resulting from the enzymatic reagents severely impede downstream applications. To improve the stability and purity of synthesized RNA, we have characterized a novel single-subunit RNA polymerase (RNAP) encoded by the psychrophilic phage VSW-3 from a plateau lake. The VSW-3 RNAP is capable of carrying out *in vitro* RNA synthesis at low temperatures (4–25°C). Compared to routinely used T7 RNAP, VSW-3 RNAP provides a similar yield of transcripts but is insensitive to class II transcription terminators and synthesizes RNA without redundant 3’-cis extensions. More importantly, through dot-blot detection with the J2 monoclonal antibody, we found that the RNA products synthesized by VSW-3 RNAP contained a much lower amount of double-stranded RNA byproducts (dsRNA), which are produced by transcription from both directions and are significant in T7 RNAP IVT products. Taken together, the VSW-3 RNAP almost eliminates both terminal loop-back dsRNA and full-length dsRNA in IVT and thus is especially advantageous for producing RNA for *in vivo* use.

## Introduction

DNA-dependent RNA polymerases (RNAPs) specifically recognize transcriptional promoter sequences and transcribe the DNA template sequence into RNA *in vivo* and *in vitro* [1]. However, for biotechnological uses that produce large amounts of RNA *in vitro*, only the single-subunit RNAPs (ssRNAPs) encoded exclusively by a group of short-tailed bacteriophages can be considered due to their simplicity and high efficiency. In the past decades, ssRNAPs from *Enterobacteria* phage T7 of the T7-like virus cluster and *Salmonella* phage SP6 of the SP6-like viruses were major enzymatic reagents for *in vitro* transcription (IVT) [2,3]. T7 RNAP has essentially dominated IVT due to extensive research and development on its use [4,5]. However, the RNA synthesized by T7 RNAP is generally contaminated with cis-extended larger products and degraded or abortive products shorter than the DNA-encoded product [6] and dsRNA byproducts from non-specific transcription initiation on non-template DNA strands [7]. The dsRNA byproducts may mimic viral genomic dsRNA and are especially harmful for *in vivo* applications [7,8]. In 2013 and 2019, we characterized representative ssRNAPs from the newly categorized P60-like marine viruses and phiKMV-like viruses, Syn5 and KP34 RNAP [9,10], respectively. These ssRNAPs enriched the IVT toolbox by demonstrating advantages over T7 RNAP in IVT, including higher processivity (Syn5) [11], insensitivity to class I T7 terminators (Syn5) [11], higher product 3’ homogeneity (KP34) [10], and a higher efficiency of incorporating modified nucleotides (Syn5) [12]. However, under current conditions, neither Syn5 nor KP34 RNAP can match the high RNA yield of T7 RNAP. These works encouraged us to further investigate novel ssRNAPs by leveraging the surge in viromes to identify natural variants that could foster innovations in rapidly progressing RNA research and medicine [13–15].

In this work, we aimed to identify an ssRNAP from a newly characterized cold-active bacteriophage that efficiently synthesized RNA at low temperatures, thus reducing the RNA degradation caused by environmental ribonuclease contamination and prolonged incubation. We targeted the *Pseudomonas fluorescens (P . fluorescens)* bacteriophage VSW-3, the genome sequence information (GB: KX066068.1) of which was released in 2017 [16]. The lytic psychrophilic bacteriophage VSW-3 was isolated together with *P. fluorescens* SW-3 cells from the Napahai Lake located in Shangri-La County in the southwest of China [17]. In the laboratory, VSW-3 phage-infected *P. fluorescens* formed 2 mm clear plaques at 4°C after 48 h [17]. The dsDNA genome of VSW-3 is 40,556 bp long and contains 46 open reading frames [17]. Together with T7, Syn5, and KP34, VSW-3 belongs to the short-tailed phage *Autographivirinae* subfamily.

Here, we successfully purified the VSW-3 RNAP (798 aa), identified its promoters, and established its *in vitro* transcription system conditions. As expected, VSW-3 RNAP efficiently produced RNA transcripts in the temperature range between 4°C and 25°C, and at 25°C, its maximum yield was comparable to that of T7 RNAP. As a natural ssRNAP, the VSW-3 RNAP is insensitive to class II transcription terminators like the T7 RNAP R173C mutant [18–20], and the RNA products of VSW-3 RNAP are free of 3’-end extensions [6,10]. Most importantly, in contrast to T7 RNAP products, dsRNA byproducts are barely detectable in VSW-3 RNAP IVT products. In addition, the Y578F mutant of VSW-3 RNAP efficiently incorporated 2’-fluoro-dATP and 2’-fluoro-dUTP into RNA [21,22].

## Materials and methods

### Materials

Oligonucleotides were synthesized by Genecreate Company, DNA purification kits were from Axygen, and Ni-NTA resin was from Qiagen. Preparative Superdex S200 for gel filtration was from GE Healthcare. The Gibson Assembly kit, T4 RNA ligase I, recombinant inorganic pyrophosphatase, rNTPs, DNase I, apyrase, T7 RNA polymerase (50 U/μL), low-range ssRNA ladder, and Monarch RNA Cleanup kit were from New England Biolabs. 2’-Fluoro-dNTPs, N6-methyl adenosine-5′-triphosphate (m6ATP), and 5-methylcytosine-5’-triphosphate (5mCTP) were from TriLink BioTechnologies. A reverse transcriptase kit and PrimeSTAR Max DNA polymerase were from TaKaRa. RiboLock RNase inhibitor and RNase A (10 mg/mL) were from Thermo Scientific. J2 monoclonal antibody (mAb) was from English & Scientific Consulting. An Immobilon-Ny+ membrane was from Millipore. X-ray film was from Kodak. DNA marker and an electrochemiluminescence (ECL) reagent were from Thermo Scientific. A high-performance liquid chromatography (HPLC) polymeric reversed-phase media (PLRP-S) column was from Agilent.

### VSW-3 RNAP expression and purification

The predicted coding sequence for VSW-3 RNAP and its Y578F mutant (Table S1) was inserted into a pCold vector harbouring an N-terminal His-tag using Gibson Assembly cloning technology [23]. After transformation, *E. coli* BL21 cells were cultured in 1 L of Luria-Bertani medium containing 100 mg/mL ampicillin at 37°C until the OD600 was approximately 0.8, at which time the incubation temperature was reduced to 10°C and 0.2 mM isopropyl β-d-1-thiogalactopyranoside was added to induce VSW-3 RNAP expression for 24 h. The cells were collected and resuspended in 25 mM Tris-HCl (pH 7.5), 300 mM NaCl, and 0.5 mM dithiothreitol (DTT) and then lysed by three freeze-thaw cycles in the presence of 0.5 mg/mL of lysozyme. The supernatant was collected after centrifugation at 18,000 g and 4°C for 1 h and filtered with a 0.45-μm filter and loaded onto a Ni-NTA agarose column pre-equilibrated with 10 volumes of elution buffer [25 mM Tris-HCl (pH 7.5) and 300 mM NaCl]. The column was washed with 10 volumes of elution buffer, first containing 20 mM imidazole and then containing 50 mM imidazole, and the majority of the His-tagged VSW-3 RNAP was eluted by elution buffer containing 100 mM imidazole. Collected eluates were concentrated to 2 mL with Ultra-15 Centrifugal Filter units (Millipore) and loaded onto a 200-mL preparative Superdex S200 column for gel filtration chromatography. Finally, VSW-3 RNAP was dialysed against buffers containing 50 mM Tris-HCl (pH 7.5), 100 mM NaCl, 1 mM DTT, 0.1 mM ethylenediaminetetraacetic acid (EDTA), 0.1% Triton X-100, and 50% glycerol and then stored at −20°C. The protein concentration of VSW-3 RNAP was determined by using a Bradford protein quantitative kit (Bio-Rad), and protein samples collected via Ni^2+^ affinity purification and gel filtration chromatography were analysed by 10% sodium dodecyl-sulphate polyacrylamide gel electrophoresis and stained with Coomassie blue (Bio-Rad).

DNase and DNA polymerase contamination in purified VSW-3 RNAP was examined in reactions containing 50 ng/μL copepod green fluorescent protein (copGFP) DNA template and 2 μM VSW-3 RNAP, in the presence or absence of a 4 mM dNTP mix in IVT buffer [40 mM Tris-HCl (pH 8.0), 16 mM MgCl_2_, 5 mM DTT, and 2 mM spermidine]. Reactions were incubated at 25°C for 12 h; then, 1 μL of 6× DNA loading buffer and 5 μL of reaction products were loaded onto a 1.5% agarose gel and electrophoresis was conducted at 100 V for 30 min. The gels were stained with ethidium bromide and visualized using a UVsolo Touch system (Analytik Jena).

### VSW-3 RNAP promoter determination

Compared to the phage genomes with known promoter sequences [10], the organization of the VSW-3 genome is most similar to that of the KP34 genome, in which both have the RNAP gene downstream of DNA metabolism genes [10]. Thus, we predicted that the sequence 5’-TTAATTGGGCCACCTATAGTA-3’ may contain the VSW-3 promoter due to its location and multiple-occurrences in the genome, with the location of KP34 promoters in the KP34 genome as a reference [10]. To reveal the exact promoter sequence and the precise transcription initiation site for VSW-3 RNAP, we first inserted the DNA fragment containing the predicted promoter sequence (5’-TTAATTGGGCCACCTATAGTA-3’) into a pUC19 plasmid between the *Bam*HI and *Xba*I sites using the Gibson Assembly method. The plasmid was linearized by *Nde*I and purified by an Axygen DNA recovery kit before serving as an IVT template.

Initially, we used the routine T7 RNAP IVT conditions for VSW-3 promoter determination. The 10 μL reaction contained 40 mM Tris-HCl (pH 7.9), 6 mM MgCl_2_, 2 mM spermidine, 1 mM DTT, 35 ng/μL of linearized pUC19 plasmid,1.5 U/μL of RNase inhibitor, 0.2 μM inorganic pyrophosphatase, 0.5 mM each of the four NTPs, and 0.2 μM VSW-3 RNAP. The IVT reaction was carried out at 20°C overnight. Then, 1 μL of DNase I was added into the reaction mixture and incubation was extended for 30 min at 37°C to remove template DNA. The transcripts (pUC19-RNA) were then purified with a Monarch RNA Cleanup kit.

The pUC19-RNA then underwent 5’-rapid amplification of cDNA ends (5’-RACE). This began with RNA 5’ monophosphorylation treatment using apyrase, according to the New England Biolabs manual. Then, 0.5 μg of monophosphorylated pUC19-RNA was ligated intermolecularly by T4 RNA ligase I in a 20 μL reaction mixture. The ligated RNA was reverse transcribed to cDNA by a reverse transcriptase (TaKaRa). A pair of primers were designed: the forward primer (5’-TCGCGCGTTTCGGTGATGACGG-3’) was located 184 nt upstream of the 3’-end of pUC19-RNA; and the reverse primer (5’-CTGATTCTGTGGATAACCGTATTAC-3’) was about 368 nt downstream of the 5’-end of pUC19-RNA. With these primers and cDNA, PCR was conducted using PrimeSTAR Max DNA Polymerase from TaKaRa. The PCR products were confirmed by agarose gel electrophoresis and inserted into a pET28a plasmid between the *Bam*HI and *Eco*RI sites using the Gibson Assembly method. The assembled plasmids were transformed into DH5α competent cells, and five colonies were picked for Sanger sequencing.

To determine the 5’ boundary of the VSW-3 RNAP promoter, we constructed dsDNA templates containing the putative VSW-3 RNAP promoter with gradual truncations at the 5’ end and the downstream 40 bp (Table S1). These dsDNA templates containing DNA with various lengths of putative VSW-3 promoters (19 bp, 18 bp, or 17 bp, Table S1) were incubated with 0, 1, 2, or 3 μM VSW-3 RNAP in IVT buffer [40 mM Tris-HCl (pH 8.0), 16 mM MgCl_2_, 5 mM DTT, and 2 mM spermidine] at 30°C for 10 min. Then, 10 μL of the mixtures were mixed with 5 μL native loading dye and analysed by 12% native Tris-borate-EDTA (TBE)-PAGE. Electrophoresis was conducted at 120 V for 60 min, and the DNA products were stained with ethidium bromide and visualized using a UVsolo Touch system. The grey values of gel bands were quantified using ImageJ software. Promoter binding percentages were calculated as the molar ratio between the bound DNA and the total DNA at each enzyme concentration.

The yield of 40 nt RNA products from the DNA templates containing the putative VSW-3 RNAP promoter of various lengths (18 bp, 17 bp, 16 bp, 15 bp, or 14 bp, Table S1) was analysed in a 10 μL reaction containing 40 mM Tris-HCl (pH 7.9), 6 mM MgCl_2_, 2 mM spermidine, 1 mM DTT, 4 μM annealed dsDNA templates, 1.5 U/μL of RNase inhibitor, 0.2 μM inorganic pyrophosphatase, 0.5 mM each of the four NTPs, and 0.2 μM VSW-3 RNAP. After incubation at 20°C overnight, 2 μL of each reaction product was mixed with 6 μL of 2x denaturing loading buffer (95% formamide, 0.02% SDS, 1 mM EDTA, 0.02% bromophenol blue, and 0.01% xylene fluoride) and 4 μL of H_2_O, heated at 85°C for 2 min, and immediately placed on ice for 2 min. Finally, 10 μL of the sample mixture was loaded onto a 12% native TBE-PAGE gel. Electrophoresis was performed at 120 V for 1 h, and the gel was stained with ethidium bromide before analysis using a UVsolo Touch system.

The efficiency of the initial nucleotide incorporation was compared to the yield of a single guide RNA (sgRNA) (coding sequence 5’-GGGCACGGGCAGCTTGCCGG GTTTTAGAGCTAGAAATAGCAAGTTAAAATAAGGCTAGTCCGTTATCAACTTGAAAAAGTGGCACCGAGTCGGTGCTTTTTTT-3’) and its variants with the 5’ first nucleotide G replaced with A, C, or T (U in RNA). The IVT reactions contained 40 mM Tris-HCl (pH 8.0), 16 mM MgCl_2_, 5 mM DTT, 2 mM spermidine, 30 ng/µL of DNA template, 1.5 U/μL of RNase inhibitor, 0.2 μM inorganic pyrophosphatase, 4 mM each of the four NTPs, and 0.2 μM of VSW-3 RNAP and were incubated at 25°C for 12 h. Then, 2 μL of denatured loading dye and 3 μL of ddH2O were mixed with 1 μL of the IVT products, and electrophoresis was conducted at 120 V for 1 h. The gels were stained with ethidium bromide and visualized using a UVsolo Touch system. The grey values of gel bands were quantified using ImageJ software.

### IVT condition screening

A pair of primers (Table S2) were designed for PCR to prepare transcription templates of *cas9* RNA from a *cas9*-coding plasmid (Addgene: 72,247, T7p-*cas9* plasmid), in which the T7 RNAP promoter was replaced with the VSW-3 RNAP promoter (VSW-3p-*cas9* plasmid). Reaction mixtures containing 0.2 μM VSW-3 RNAP, 35 ng/μL of *cas9* DNA template, 40 mM Tris-HCl (pH 8.0), 2 mM spermidine, 1.5 U/μL of RNase inhibitor, 0.2 μM inorganic pyrophosphatase, and various concentrations of MgCl_2_ (0 mM, 1 mM, 2 mM, 3 mM, 4 mM, 5 mM, 6 mM, 7 mM, 8 mM, 9 mM, 10 mM, 12 mM, 14 mM, 16 mM, 18 mM, or 20 mM) in combination with various concentrations of NTPs (0.5 mM, 1 mM, 2.5 mM, 4 mM, or 5 mM) were screened for optimal RNA yields. DTT concentrations (1 mM, 5 mM, or 20 mM) were also screened to identify a stable and high-yielding transcription buffer with optimal MgCl_2_ and NTP concentrations.

Using the optimal IVT conditions [40 mM Tris-HCl (pH 8.0), 16 mM MgCl_2_, 5 mM DTT, 2 mM spermidine, 4 mM NTPs, 35 ng/µL of *cas9* DNA template, 1.5 U/μL of RNase inhibitor, and 0.2 μM inorganic pyrophosphatase], we screened various enzyme concentrations (0.001 μM, 0.003 μM, 0.01 μM, 0.03 μM, 0. 1 μM, 0.15 μM, or 0.3 μM) at 20°C overnight for optimal RNA yield. Finally, we screened incubation temperatures (4°C, 10°C, 15°C, 20°C, 25°C, 30°C, or 37°C) in combination with various incubation times in the presence of 0.15 μM VSW-3 RNAP for maximal RNA yield.

After IVT, 1 μL of reaction mixture was added directly into 3 μL of 2x denaturing loading buffer (95% formamide, 0.02% SDS, 1 mM EDTA, 0.02% bromophenol blue, and 0.01% xylene fluoride) and 2 μL of H_2_O, heated at 85°C for 2 min, and immediately placed on ice for 2 min. Then, 5 μL of each sample was loaded onto a 1.5% TAE agarose gel for electrophoresis (100 V, 30 min). The gel was stained with ethidium bromide, and gel imaging was analysed using a UVsolo Touch system.

### Transcription termination

A group of terminated RNA products estimated to be 1500–1600 nt in length were observed in the T7 RNAP products during *cas9* RNA synthesis but not in the VSW-3 RNAP products. To find the accurate termination site of the T7 RNA product, a 3’-RACE test was conducted. First, copGFP-RNA synthesized using VSW-3 RNAP was used as an adapter after monophosphorylation treatment with apyrase. Then, 0.5 μg of monophosphorylated copGFP-RNA was ligated to the 3’-end of 0.5 μg of *cas9* transcripts with T7 RNAP in a 20 μL reaction mixture using T4 RNA ligase I. After reverse transcription with random primers, the cDNA was amplified in a PCR reaction with a forward primer (5’-GTATTGCCTAAGCACAGTTTACT −3’, 1533 nt downstream of the 5‘-end of *cas9* RNA), reverse primer (5’-TAGCCCATCACGTGGCTCAGCA-3’, 187 nt downstream of the 5’-end of copGFP-RNA adapter), and PrimeSTAR Max DNA Polymerase (TaKaRa). The PCR products were verified by agarose gel electrophoresis and then inserted into a pET28 plasmid between the *Bam*HI and *Eco*RI sites with the Gibson Assembly method. Plasmids were then transformed into DH5α competent cells, and five colonies were picked for Sanger sequencing.

To confirm that the VSW-3 RNAP was not sensitive to class II terminators, we inserted a class II terminator site, 5’-ATCTGTT-3’, into the copGFP RNA coding sequence located 433 nt downstream of the 5’-end. The 10 μL IVT reaction mixture contained 40 mM Tris-HCl (pH 8.0), 16 mM MgCl_2_, 5 mM DTT, 2 mM spermidine, 35 ng/μL of DNA template, 1.5 U/μL of RNase inhibitor, 0.2 μM inorganic pyrophosphatase, 4 mM each of the four NTPs, and 0.15 μM VSW-3 RNAP. After IVT, the RNA products were assayed by 1.5% agarose gel electrophoresis, as described in previous sections.

### Synthesis of sgRNA

DNA fragments containing the coding sequence of an sgRNA (5’-GGGCACGGGCAGCTTGCCGG GTTTTAGAGCTAGAAATAGCAAGTTAAAATAAGGCTAGTCCGTTATCAACTTGAAAAAGTGGCACCGAGTCGGTGCTTTTTTT-3’) targeting the eGFP gene under the control of a T7 or VSW-3 RNAP promoter were inserted into a pUC19 plasmid between the *Bam*HI and *Xho*l sites. Then, a pair of universal amplification primers (sgRNA template-F: 5’-ATCAGGCGCCATTCGCCATTCAGG-3’ and sgRNA template-R: 5’- AAAAAAAGCACCGACTCGGTGCCACT-3’) were used for PCR amplification of the sgRNA IVT product. PCR products were cleaned with a DNA purification kit (Axygen). The 10 μL IVT reaction mixture contained 40 mM Tris-HCl (pH 8.0), 16 mM MgCl_2_, 5 mM DTT, 2 mM spermidine, 35 ng/µL of VSW-3p-sgRNA or T7p-sgRNA template DNA, 1.5 U/μL RNase inhibitor, 0.2 μM inorganic pyrophosphatase, 4 mM each of the four NTPs, and either 0.15 μM of VSW-3 RNAP (25°C for 12 h) or T7 RNAP (37°C for 1 h). After IVT, the RNA products were analysed by 12% TBE-PAGE, as described in previous sections.

3’-RACE was carried out to verify the sgRNA product terminal homogeneity from the T7 and VSW-3 RNAP IVTs. Monophosphorylated copGFP RNA was ligated to the 3’-end of T7 and VSW-3 sgRNA transcripts with RNA ligase I. After reverse transcription, cDNA was amplified by PCR (primers: 3’ RACE-sgRNA-F: 5’-GCAGCTTGCCGGGTTTTAGAGCTAG-3’ and 3’ RACE-sgRNA-R: 5’-TAGCCCATCACGTGGCTCAGCA-3’). The PCR products were verified by 1.5% agarose gel electrophoresis and inserted into a pET28 plasmid between the *Bam*HI and *Eco*RI sites with the Gibson Assembly method. Ten colonies from each group were selected for Sanger sequencing. Additionally, 3’-RACE was also performed to verify the terminal homogeneity of the long RNA product (eGFP RNA, Table S2) from the T7 and VSW-3 RNAP IVTs, and five colonies from each group were selected for Sanger sequencing.

The sgRNA products synthesized by VSW-3 RNAP and T7 RNAP were purified using a Monarch RNA Cleanup kit (New England Biolabs) and then further purified by HPLC using a PLRP-S column (4000 A, 8 μM, 4.6 × 150 mm). To confirm that the origin of the sgRNA 3’ extension was from the RNA-dependent RNA polymerase (RdRp) activity of T7 RNAP [24], the HPLC-purified VSW-3 sgRNA products were used as the RdRp template for T7 RNAP and VSW-3 RNAP (final sgRNA concentration of 0.5 μg/μL). Other components in the reaction mixture were as follows: 40 mM Tris-HCl (pH 8.0), 16 mM MgCl_2_, 5 mM DTT, 2 mM spermidine, 4 mM each of the four NTPs, 0.2 μM inorganic pyrophosphatase, 1.5 U RNase inhibitor, and either 0.15 μM T7 RNAP (37°C for 1 h) or VSW-3 RNAP (25°C for 12 h). Reaction products were analysed by 12% TBE-PAGE, as described in a previous section.

### Incorporation of modified nucleotides

To improve the resistance of the RNA to RNases, 2’-fluoro (F)-dNTPs were incorporated into the RNA *in vitro* and *in vivo* [21,22]. Replacement of a tyrosine with a phenylalanine in T7 RNAP, Y639F [25], Syn5 RNAP Y564F [12], and KP34 RNAP Y603F [10] allowed the synthesis of partially modified 2’-F-RNA. Through homologous sequence alignment of VSW-3 RNAP, T7 RNAP, Syn5 RNAP, and KP34 RNAP with Geneious software, we identified the equivalent amino acid Y578 in VSW-3 RNAP, constructed a VSW-3 RNAP Y578F mutant, and purified the mutant enzyme using the same procedure used for the wild-type (WT) VSW-3 RNAP. Efficiency of the WT and Y578F VSW-3 RNAP to incorporate four 2’ -F-dNTPs and other modified nucleotide substrates was tested in reactions containing 40 mM Tris-HCl (pH 8.0), 16 mM MgCl_2_, 5 mM DTT, 2 mM spermidine, 1.5 U/μL of RNase inhibitor, 0.2 μM inorganic pyrophosphatase, 4 mM each of the four NTPs (with one of the four NTPs replaced by their 2’-F-dNTP analogue, m6ATP (replacing ATP) or 5mCTP (replacing CTP)), either 0.15 μM of VSW-3 RNAP or the VSW-3 RNAP-Y578F mutant, and 35 ng/μL of the VSW-3p-sgRNA template DNA. Reactions were carried out at 25°C for 12 h, and products were analysed by 12% TBE-PAGE as described in previous sections.

### Dot-blot detection of dsRNA in IVT transcripts

We conducted dot-blot tests with a J2 monoclonal antibody to detect dsRNA in T7 and VSW-3 RNAP IVT transcripts (*SOX7*, tdTomato (from pUCP20T-tdTomato), copGFP and *cas9* RNA) (Table S3). The *cas9* coding sequences of the T7p-*cas9* and VSW-3p-*cas9* plasmids were replaced by either the *SOX7*, tdTomato, or copGFP gene coding sequences. The transcription templates for *SOX7* and tdTomato RNA were plasmid DNA linearized with *BspQ*I restriction endonuclease; the transcription templates for copGFP and *cas9* RNA were prepared by PCR amplification with primers Trans_Template-cas9-F and Trans_Template-cas9-R (Table S1). *SOX7* RNA (1388 nt), tdTomato RNA (1689 nt), copGFP RNA (918 nt), and *cas9* RNA (4258 nt) were transcribed in reactions containing 40 mM Tris-HCl (pH 8.0), 16 mM MgCl_2_, 5 mM DTT, 2 mM spermidine, 1.5 U/μL of RNase inhibitor, 0.2 μM inorganic pyrophosphatase, 4 mM each of the four NTPs, 35 ng/µL of template DNA, and either 0.15 μM of VSW-3 RNAP (25°C for 12 h) or T7 RNAP (37°C for 1 h). After IVT, DNA templates were removed by DNase I and transcripts were purified using a Monarch RNA Cleanup kit.

dsRNA (0.1 ng, 0.25 ng, 0.5 ng, and 1.0 ng) used as a quantitative standard and 200 ng of the *SOX7*, tdTomato, copGFP, and *cas9* RNA transcripts were dropped onto an Immobilon^TM^ -Ny+ Membrane (Millipore), dried, blocked with 5% non-fat dry milk in TBS-T buffer [50 mM Tris-HCl (pH 7.4), 150 mM NaCl, and 0.05% Tween-20], and incubated with the dsRNA-specific J2 mAb (English & Scientific Consulting) for 30 min at 25°C. The membranes were washed two times with TBS-T buffer, reacted with hydrogen peroxidase-conjugated donkey anti-mouse Ig (Jackson Immunology), washed two times, and then detected with an ECL detection reagent (Thermo). The ECL signals were captured on an X-ray film (Kodak) through photosensitive development.

In addition, copGFP RNA synthesized by VSW-3 RNAP was used as a template (to replace the DNA template) in T7 and VSW-3 IVTs, and the products were compared to the untreated copGFP RNA and copGFP dsRNA (by annealing copGFP RNA with its complementary copGFP RNA (-) (Table S2) in 5x RNA annealing buffer (Beyotime, #R0051)) using 1.5% agarose gel electrophoresis and dot-blot analysis, as described in a previous section.

## Results and discussion

### Psychrophilic bacteriophage VSW-3 RNAP and its promoter

**Figure 1.**
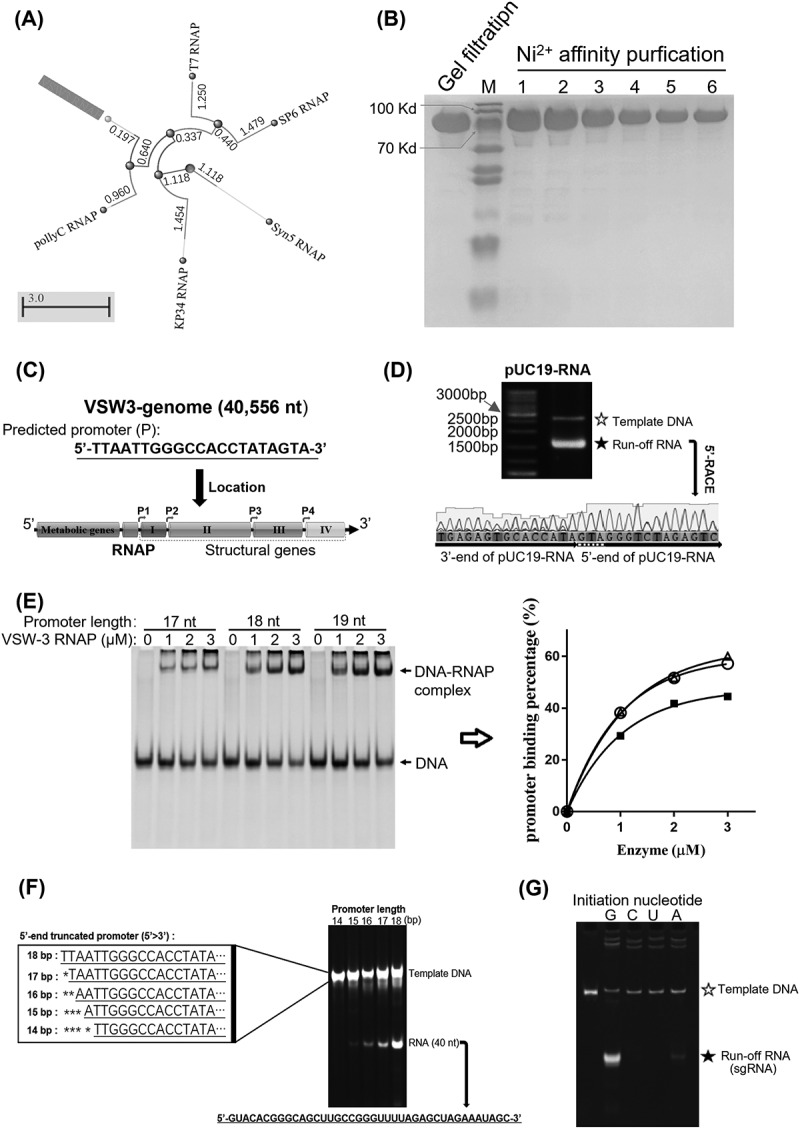
VSW-3 RNAP and its promoter. (A) Distance tree analysis of the representative ssRNAPs by the BLAST program. Distance from the root ‘○’: SP6 RNAP (3.374) > T7 RNAP (3.145) > KP34 RNAP (2.572) > VSW-3 RNAP (2.292) > Syn5 RNAP (1.118) suggests that VSW-3 RNAP is the second primitive after Syn5 RNAP and evolved into a new branch of the evolutionary tree together with a predicted PollyC RNAP (3.055) from phage PollyC (YP_009622558.1). (B) SDS-PAGE analysis of purified VSW-3 RNAP (92.4 kDa). (C) Organization of phage VSW-3 genome and distribution of the predicted VSW-3 promoters (indicated by right arrows). (D) IVT of VSW-3 RNAP on the linearized pUC19 plasmid with an insertion of the predicted VSW-3 promoter (top gel). 5’-RACE revealed that the initial nucleotide of VSW-3 RNAP transcription in the predicted promoter was 5’-GTA-3’ (bottom sequencing result). (E) Binding of DNA containing various lengths of putative VSW-3 promoters (17 bp: 5’-TAATTGGGCCACCTATA-3’; 18 bp: 5’-TTAATTGGGCCACCTATA-3’; and 19 bp: 5’-TTTAATTGGGCCACCTATA-3’) by VSW-3 RNAP in IVT buffer at 30°C for 10 min. Binding mixtures were analysed by 12% native TBE–PAGE. The grey values of gel bands were quantified using ImageJ software. Promoter binding percentages were calculated as the molar ratio between bound DNA and total DNA at each enzyme concentration. The binding curve on the right panel was generated by GraphPad Prism. The filled square indicates DNA binding with the 17 bp promoter, the empty triangle indicates DNA binding with the 18 bp promoter, and the empty circle indicates DNA binding with the 19 bp promoter. (F) IVT on 5’-truncated DNA templates (left box) to determine the specific promoter of VSW-3 RNAP. The RNA yield with each template (right gel) suggested that the 15 bp (5’-ATTGGGCCACCTATA-3’) sequence is the minimal promoter, and the 18 bp (5’-TTAATTGGGCCACCTATA-3’) sequence is the full VSW-3 promoter. (G) Comparison of the VSW-3 IVT yield of an sgRNA and its variants with the 5’ first ‘G’ replaced by a ‘C’, a ‘U’, or an ‘A’, respectively, as indicated at the top of the gel.

The newly discovered VSW-3 RNAP is an ssRNAP from Napahai Lake in the southwestern plateau of China with an average annual temperature of 5°C. It has a homology of 31% to T7 RNAP, and the distance tree analysis revealed that VSW-3 RNAP has a different evolutionary distance and direction compared with T7, SP6, Syn5, or KP34 RNAP (Fig. 1A). As the first RNAP characterized from cold-active bacteriophage, we successfully expressed VSW-3 RNAP at 10°C. VSW-3 RNAP is smaller in size than T7 RNAP (798 aa vs. 883 aa). The SDS-PAGE analysis revealed that the size of His-tagged VSW-3 RNAP is consistent with the protein size prediction (92.4 kDa), with protein purity over 90% after nickel column and gel filtration chromatography (Fig. 1B). No DNase or DNA polymerase activity was detected in high concentrations of purified VSW-3 RNAP in the IVT system (Figure S1).

For bacteriophage T7 and SP6, transcription initiation mainly depends on a 17 bp promoter sequence that appears several times in their genomes [26–28]. However, for Syn5 and KP34 [9,10], there are only a few promoters across their genomes, making promoter prediction difficult. Based on our previous summary of the transcription promoter distribution of representative short-tailed phages, in which there is at least one phage promoter between the RNAP gene and the following open reading frame [10] and the existence of the conservative TATA boxes [29], we predicted that a 21-bp nucleotide sequence (5’-TTAATTGGGCCACCTATAGTA-3’) that appeared four times in the VSW-3 genome most likely harboured the VSW-3 promoter. One of the predicted promoter regions followed the VSW-3 RNAP gene (13,579–13,599) and the other three, located at 17,122–17,142, 28,006–28,026, and 34,846–34,866, were all in intergenic regions (Fig. 1C).

Using a linearized pUC19 plasmid with the predicted VSW-3 promoter inserted as a transcription template, the IVT activity of VSW-3 RNAP was demonstrated (Fig. 1D). We analysed the IVT transcripts to confirm the VSW-3 RNAP transcriptional initiation site, and 5’-RACE revealed that the transcripts started with ‘GTA’ (Fig. 1D). DNA binding analysis indicated that the 18 bp sequence (5’-TTAATTGGGCCACCTATA-3’) was the full promoter for VSW-3 RNAP, as one nucleotide removed at the 5’ end (17 bp sequence) weakened the binding by VSW-3 RNAP, while a 5’ extension (19 bp sequence) did not enhance the binding (Fig. 1E). The IVT yield of a 40 nt RNA under the control of the VSW-3 promoter of various lengths revealed that a 15 bp sequence (5’-ATTGGGCCACCTATA-3’) was the minimal promoter and the 18 bp sequence (5’-TTAATTGGGCCACCTATA-3’) was the full promoter for VSW-3 RNAP (Fig. 1F). The main difference between the T7 (5’-TAATACGACTCACTATA-3’) and VSW-3 promoters was the middle 8 bp sequence (T7: 5’-ACGACTCA-3’; VSW-3: 5’-TGGGCCAC-3’). Similar to that of T7 RNAP, VSW-3 RNAP initiated RNA synthesis efficiently with GTP, less efficiently with ATP, and was unable to use CTP or UTP as an initiation nucleotide (Fig. 1G).

### Optimized VSW-3 RNAP IVT system

**Figure 2.**
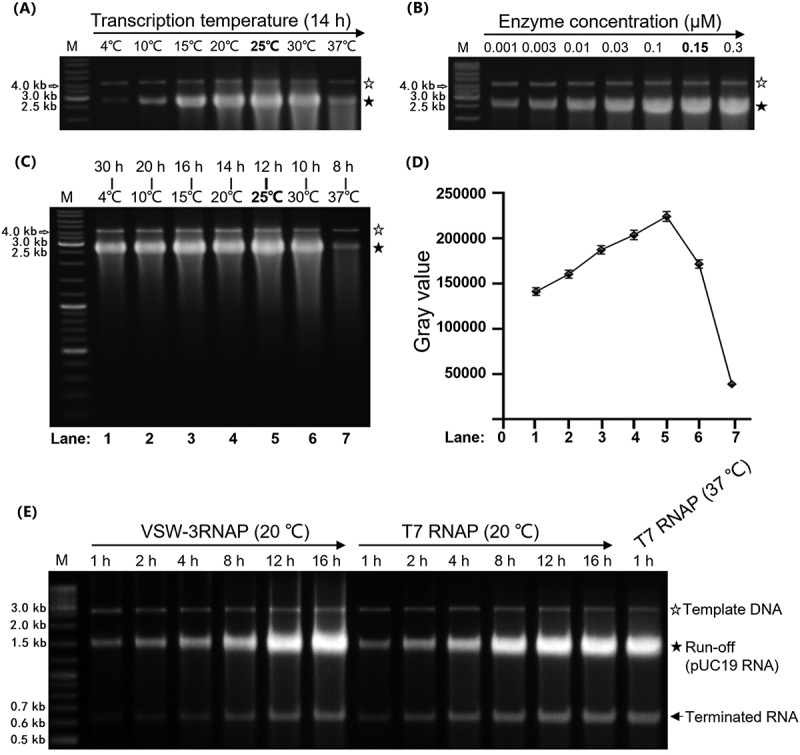
VSW-3 RNAP IVT conditions. (A) Optimal reaction temperature of the VSW-3 RNAP IVT (25°C) for maximum run-off *cas9* RNA yield. (B) Optimal enzyme concentration of the VSW-3 RNAP (0.15 μM) IVT for maximum run-off *cas9* RNA yield. (C) Optimal IVT yield of VSW-3 RNAP with various reaction temperature and incubation time combinations. The maximum run-off *cas9* RNA yield was obtained at 25°C for 12 h. (D) Gray scale quantification of the run-off RNA transcripts in a gel **(C)**. The diagram was made using GraphPad Prism. (E) Optimal pUC19-RNA yield by VSW-3 RNAP was obtained at 20°C for 16 h, compared to the optimal yield of the same RNA by T7 RNAP at 20°C for 16 h and at 37°C for 1 h, under the same IVT conditions. In all gels, the bands corresponding to DNA templates are indicated by empty stars, and the bands corresponding to run-off RNA transcripts are indicated by filled stars. An arrow indicates terminated transcripts caused by a class I T7 terminator.

We found that the IVT yield of VSW-3 RNAP was significantly affected by the concentration of Mg^2+^ and DTT, and higher concentrations of NTPs required a corresponding higher Mg^2+^ concentration in the transcription buffer (Figure S2A). Although the buffer containing 9 mM Mg^2+^ and 1 mM DTT supported an optimal yield of *cas9* RNA in the presence of 4 mM of NTPs (Figure S2A), it was unstable and the yield of *cas9* RNA dropped significantly when the buffer was stored at −20°C for a few days (Fig. 2A). Higher concentrations of DTT (5 mM and 20 mM) increased the buffer stability and also required higher Mg^2+^ concentrations to reach the maximum yield of RNA (Figure S2B). Finally, we established an efficient and stable (more than 6 months) IVT buffer for VSW-3 RNAP [40 mM Tris-HCl (pH 8.0), 16 mM MgCl_2_, 5 mM DTT, and 2 mM spermidine].

VSW-3 RNAP produced RNA over a wide range of temperatures (4–37°C) *in vitro*, with the highest yield at 25°C (Fig. 2A). At lower temperatures, the yield increased as the incubation time was extended (Figure S2C). The optimal enzyme concentration of VSW3 RNAP was 0.15 μM (Fig. 2B), similar to that of T7 RNAP. At 37°C, which is the optimal temperature for T7 RNAP, the yield of VSW-3 RNAP was even lower than the yield at 4°C (Fig. 2C). Maximum IVT yields of RNA for VSW-3 RNAP were obtained at 25°C after a 12-h incubation (Fig. 2C and 2D). A working stock of 1.5 μM VSW-3 RNAP was stable for more than 6 months at −20°C.

**Figure 3.**
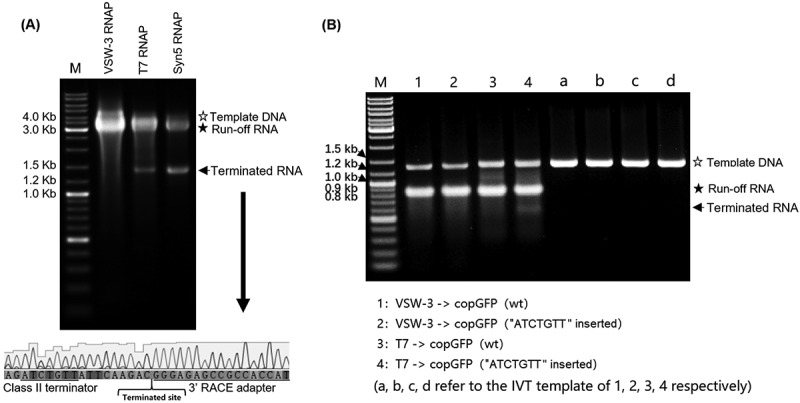
Response of ssRNAPs to the class II T7 terminator. (A) Using PCR-amplified templates for *cas9* RNA IVT, obvious abortive RNA transcripts were observed for T7 RNAP and Syn5 RNAP but not for VSW-3 RNAP (left gel). 3’-RACE revealed that the T7 RNAP transcription was terminated 9 nt downstream of a class II terminator 5’-ATCTGTT-3’ (bottom sequencing result). (B) Insertion of a class II T7 terminator 5’-ATCTGTT-3’ into the coding sequence of copGFP RNA caused termination of T7 RNAP but not of VSW-3 RNAP. In all gels, the bands corresponding to DNA templates are indicated by empty stars, and the bands corresponding to run-off RNA transcripts are indicated by filled stars. Arrows indicate terminated transcripts caused by class II T7 terminators.

With the same optimal buffer and concentrations of enzyme and substrates, the maximum yields of VSW-3 (25°C, 12 h) and T7 RNAP (37°C, 1 h) were comparable, while for the synthesis of long RNAs such as *cas9* RNA (Fig. 3A), tdTomato RNA (GenBank: KT878736.1), and the complete S protein RNA of SARS-CoV-2 (GenBank: NC_045512.2), the optimal yield of VSW-3 RNAP (quantified as about 70 μg per 20 μL IVT reaction for these RNAs) was higher than that of T7 RNAP (quantified as about 60 μg per 20 μL IVT reaction for these RNAs).

The optimized transcription buffer for VSW-3 RNAP (16 mM MgCl_2_ and 5 mM DTT) was also optimal for T7 RNAP to achieve high yields in the presence of 4 mM NTPs, compared to the routine buffer (6 mM MgCl_2_ and 1 mM DTT, New England Biolabs) (Figure S3A and S3B). Under this condition, the T7 RNAP reached its maximum yield in 1 h, and further extending the incubation did not increase the yield but decreased the amount of run-off products (Figure S3A and S3B). We also compared the pUC19-RNA yield of VSW-3 and T7 RNAP IVTs at 20°C (thus, all IVT conditions were the same for each enzyme) and consistently found that the maximum yield of VSW-3 RNAP IVT was comparable to that of T7 RNAP IVT (Fig. 2E).

### Transcription termination

During the production of pUC19-RNA (Table S3), we observed a byproduct shorter than the run-off RNA for both T7 and VSW-3 RNAP (Figure S3A). 3’-RACE of the terminus of the shorter RNA and structural analysis of the upstream sequence revealed that this byproduct was due to the transcription termination by both enzymes at a T7 class I [18–20] terminator-like stem-loop structure (Figure S3C and S3D, Table S3 red sequence).

There are two classes (class I and class II) of transcription terminators known to partially stop the transcriptional elongation of T7 RNAP. The class I terminator forms a stem-loop structure, and the class II terminator typically contains a 7 nt sequence (5’-ATCTGTT-3’) [18,20,30]. During the transcriptional assays with PCR product as the transcription template for *cas9* RNA (Addgene: 72,247), we found that there was a group of terminated RNA products synthesized by T7 and Syn5 RNAP. According to the markers in the agarose gel, the terminated RNA products were estimated to be 1500–1600 nt in length (Fig. 3A gel). By 3’-RACE, we found that the termination site of T7 RNAP was located 9 nt downstream of a canonical class II terminator ‘ATCTGTT’ in the *cas9* RNA (Fig. 3A bottom). However, in the same RNA, this terminator showed no effect on VSW-3 RNAP (Fig. 3A), and A class II terminator 5’-ATCTGTT-3’ inserted into the copGFP gene did not cause any termination by VSW-3 RNAP (Fig. 3B).

### 3’ termini of RNA products

**Figure 4.**
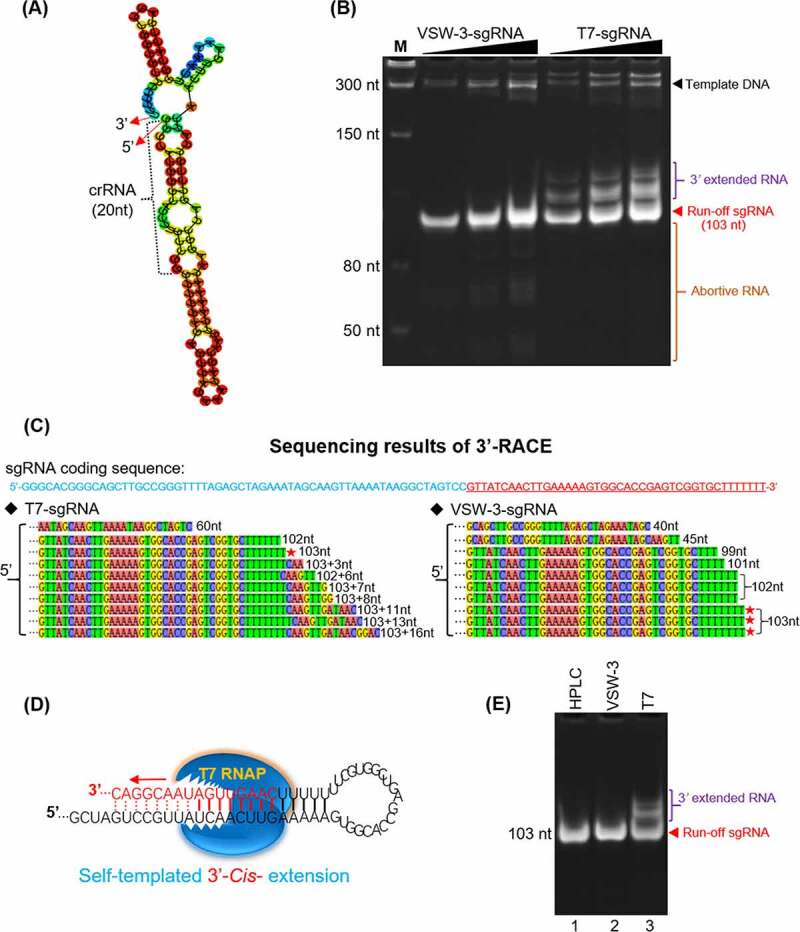
RNA 3’ extension and RdRp activity of T7 and VSW-3 RNAP. (A) Secondary structure of an sgRNA predicted with RNAfold software. (B) IVT synthesis of an sgRNA (targeting eGFP) by VSW-3 and T7 RNAP. (C) 3’-RACE of the sgRNA transcripts from T7 and VSW-3 RNAP IVTs. Only the 3’ region (red sequence on the top) of the full sgRNA in the sequencing results is shown. The length of each sequence is noted. The sequences matching the exact run-off sgRNA (103 nt) are indicated by red stars. (D) Schematic showing the mechanism and origin (3’ self-templated extension by the RdRp activity of T7 RNAP) of the 16 nt 3’-extension in T7 RNAP products as in **(C)**. (E) T7 but not VSW-3 RNAP retained RdRp activity to extend purified sgRNA (with terminal primer-template structure).

T7 RNAP retains RdRp activity [24] and catalyses the self-templated extension on the RNA 3’ hairpin structure [6,31–33]. The recently widely used sgRNA (103 nt) is a typical RNA with a terminal secondary structure. The first 20 nt sequence of the sgRNA tested in this work is a CRISPR RNA (5’-GGGCACGGGCAGCTTGCCGG-3’) targeting eGFP, and the remaining 83 nt sequence is the guide RNA backbone. The secondary structure of this sgRNA was predicted with RNAfold [34] (Fig. 4A). With a native 12% TBE-PAGE assay, products with extended 3’ termini were observed for T7 RNAP but not for VSW-3 RNAP, although the latter produced more abortive products (Fig. 4B). 3’-RACE revealed that among the 10 colonies picked for T7 RNAP sgRNA product sequencing (Fig. 4C), only one sequencing result matched the sgRNA (103 nt), and seven sequencing results revealed 3’ extensions up to 16 nt. The mechanism by which the RdRp activity of T7 RNAP creates these extensions is illustrated in Fig. 4D. The parallel sequencing results for the VSW-3 RNAP sgRNA products showed no 3’ extension, consistent with the gel analysis (Fig. 4B). Out of the 10 sequences, three matched the exact sgRNA sequence (103 nt), three had one missing nucleotide (102 nt), and the other four showed further truncations (101 nt, 99 nt, 40 nt, and 33 nt), for which the underlying mechanism is unclear (Fig. 4C).

We examined the RdRp activity of T7 and VSW-3 RNAP that used the HPLC-purified sgRNA product of VSW-3 RNAP as a template (Fig. 4E). Activity was not detected for VSW-3 RNAP as the sgRNA template with a terminal loop-back dsRNA structure was not extended. However, T7 RNAP efficiently extended the RNA template with its RdRp activity (Fig. 4E), confirming the origin of the 3’ terminal loop-back dsRNA region in the T7 RNAP IVT products.

We also examined the 3’-termini of long IVT products. Among the five colonies picked for both VSW-3 RNAP and T7 RNAP sequencing following 3’-RACE, only one sequencing result matched the actual eGFP RNA sequence (978 nt) for both VSW-3 RNAP and T7 RNAP (Figure S4A). VSW-3 RNAP produced eGFP RNA transcripts shortened by one or more nucleotides (Figure S4B), while T7 RNAP consistently produced transcripts with an extension of one or more 3’ nucleotides (Figure S4C and S4D).

The self-templated 3’-extension of the T7 IVT products resulted in the formation of the loop-back dsRNA (Fig. 4D and S4), which was not formed in the VSW-3 IVT due to the lack of RdRp activity of VSW-3 RNAP.

### VSW-3 RNAP mutant for incorporation of modified nucleotides

**Figure 5.**
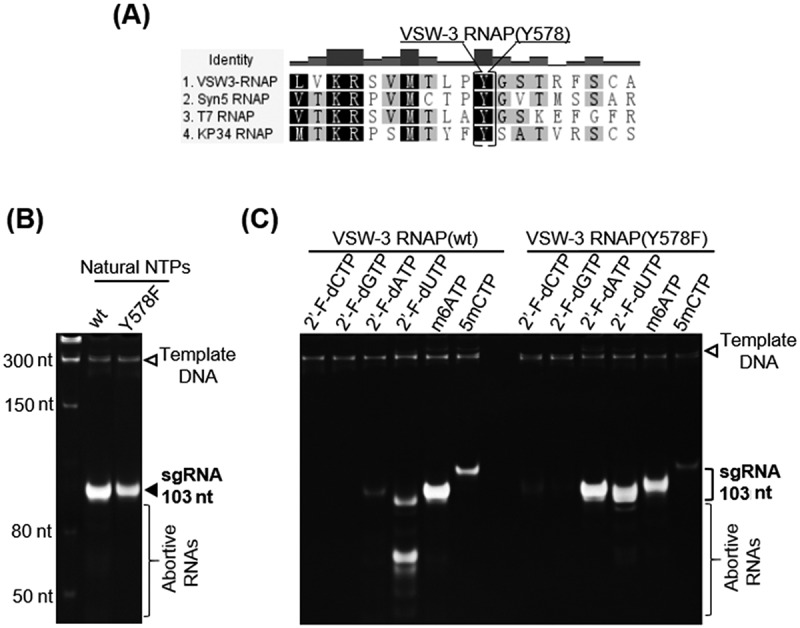
Incorporation of modified nucleotides by WT and Y578F VSW-3 RNAPs. (A) Homologous sequence alignment of VSW-3 RNAP, T7 RNAP, Syn5 RNAP, and KP34 RNAP using Geneious software identified the tyrosine (Y578) in the O-helix region of the VSW-3 RNAP. (B) IVT yield of an sgRNA by WT and Y578F VSW-3 RNAPs was compared. (C) In the IVT system for sgRNA synthesis, the incorporation of modified nucleotides by WT and Y578F VSW-3 RNAP was compared. In each reaction, one of the four NTPs was replaced by its modified analogue (as indicated at the top of the gel). The efficiency of incorporation was judged based on the yield of the run-off sgRNA.

ssRNAPs usually prefer NTPs over dNTPs and 2’-F-dNTPs; however, the replacement of a tyrosine with a phenylalanine in an O-helix region, such as those in T7 (Y639F) [25,35], Syn5 (Y564F) [12], and KP34 (Y603F) [10] RNAP mutants, weakens this discrimination and allows modified 2’-F-RNA synthesis. RNA containing ribose modifications such as 2’-F is more resistant to RNase A, which recognizes the 2’-OH of pyrimidines for cleavage, resulting in longer survival times of 2’-F-RNA *in vitro* and *in vivo* [21,22,36,37]. We investigated the effect of this mutation in the VSW-3 RNAP. Homologous sequence alignment revealed the equivalent amino acid Y578 in VSW-3 RNAP (Fig. 5A). The Y578F mutation decreased the yield of native RNA (Fig. 5B). When UTP in the sgRNA IVT reaction was replaced by 2’-F-dUTP, the WT VSW-3 RNAP produced small amounts of run-off products along with a large number of abortive products, indicating that the 2’-F-dUTP decreased the processivity of VSW-3 RNAP (Fig. 5C). This effect was eliminated by the Y578F mutation as the mutant efficiently produced run-off sgRNA with 2’-F-dUTP (Fig. 5C). Unexpectedly, the incorporation of 2’-F-dCTP was only slightly improved by the VSW-3 Y578F mutation. Instead, the incorporation of 2’-F-dATP was significantly increased by the VSW-3 Y578F mutation (Fig. 5C), unlike the KP34 RNAP Y603F and Syn5 RNAP Y564F mutants [37]. Neither the WT nor the Y578F VSW-3 RNAP incorporated 2’-F-dGTP. We also examined the incorporation of other modified nucleotides including N6-methyl-ATP (m6ATP) and 5-methyl-CTP (5mCTP), and both the WT and Y578F VSW-3 RNAP efficiently incorporated m6ATP but inefficiently utilized 5mCTP (Fig. 5C).

### Full-length dsRNA by-products

**Figure 6.**
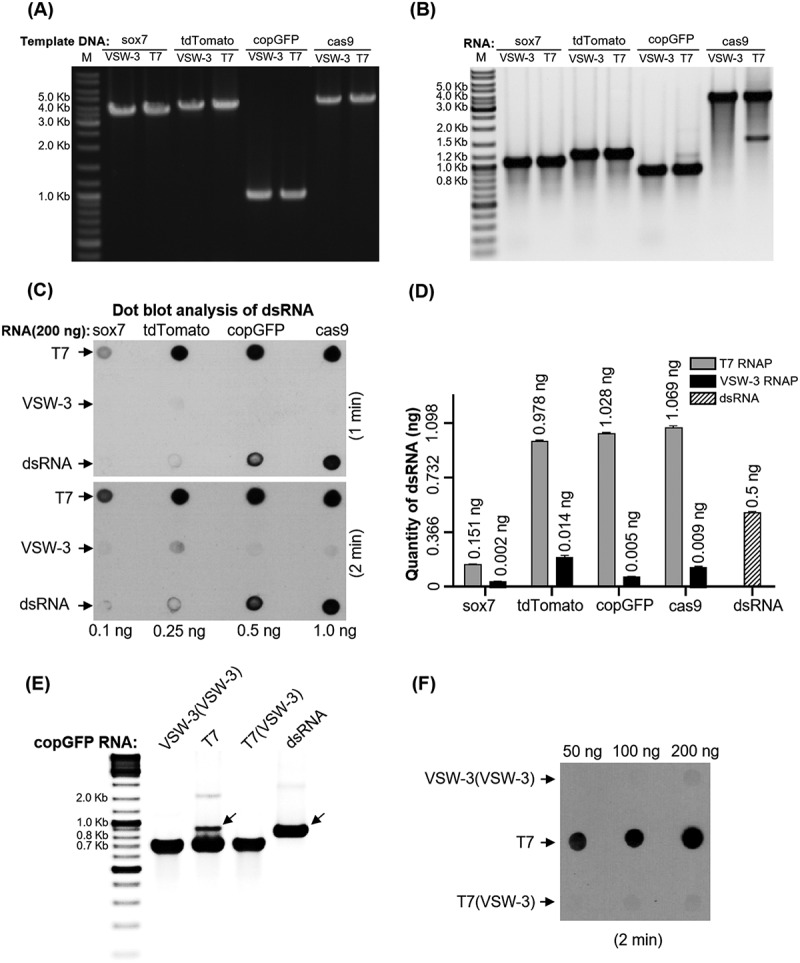
Full-length dsRNA byproducts from T7 and VSW-3 RNAP IVTs. (A) DNA templates for the IVT synthesis of various RNAs, as indicated at the top of the gel. For each RNA, there were two DNA templates differing only in the promoter region to serve for either VSW-3 or T7 RNAP IVTs. DNA concentration and purity were compared using a 1.5% agarose gel stained with ethidium bromide. (B) After template DNA was removed by DNase I treatment and purified using a Monarch RNA Cleanup kit, 1 μg of *SOX7*, tdTomato, copGFP, and *cas9* RNA transcribed by VSW-3 RNAP or T7 RNAP were analysed on a 1.5% agarose gel stained with ethidium bromide. The white and black colours for bands and background were converted in this gel image to make the weaker double-stranded and abortive RNA bands clearer. (C) Dot-blot analysis of the RNA products (each 200 ng) as in **(B)** by VSW-3 RNAP and T7 RNAP with J2 monoclonal antibody. A prepared dsRNA (351 bp) was applied as a quantitative standard (0.1 ng, 0.25 ng, 0.5 ng, 1.0 ng). (D) Gray value measurement and calculation of the X-ray film image (top image in **(C)**) by ImageJ software demonstrating the level of dsRNA contamination in T7 and VSW-3 RNAP transcripts. (E) Gel analysis of the VSW-3 (VSW-3) RNA (products of VSW-3 RNAP IVT with the purified VSW-3-copGFP RNA as the template), T7-copGFP RNA, T7 (VSW-3) RNA (products of T7 RNAP IVT with the purified VSW-3-copGFP RNA as the template), and annealed copGFP dsRNA (black arrow), with no additional band corresponding to dsRNA observed for VSW-3 (VSW-3) RNA and T7 (VSW-3) RNA. (F) Dot-blot analysis of VSW-3 (VSW-3) RNA and T7 (VSW-3) RNA showed that no dsRNA product signal was detected, confirming that full-length dsRNA products did not originate from RNA-templated RdRp activity.

Full-length dsRNA is another routine byproduct from T7 RNAP IVT [7,8], resulting from annealing of the desired plus-strand RNA transcripts with the complementary minus-strand RNA transcripts. T7 RNAP non-specifically recognizes the terminal structures in both ends of the DNA template to initiate transcription and produces minus-strand complementary RNA transcripts [7]. It is difficult to remove the full-length dsRNA completely through purification, even by HPLC, especially from scaled-up IVT products [5]. Furthermore, the remaining full-length dsRNA, although in small amounts, may cause severe cellular immune responses by mimicking RNA virus genome components, if the RNA transcripts are applied *in* vivo [7,8,38]. In some T7 RNAP IVT products, the full-length dsRNA can be observed on agarose gel as slower moving bands larger than the major run-off RNA transcripts, as in the case of copGFP (GenBank: KX757255.1, Table S3) RNA synthesis (Fig. 6B). However, for the same RNA, the band corresponding to the full-length dsRNA was not observed for the VSW-3 RNAP IVT (Fig. 6B). For other transcripts, such as *SOX7* (GenBank: NM_031439.4, Table S3), tdTomato (GenBank: KT878736.1, Table S3), and *cas9* RNA (Addgene: 72,247, Table S3), although the full-length dsRNA from the T7 RNAP IVT was not obvious on the gel (Fig. 6B), the dot-blot assay with the J2 mAb confirmed that for all of the T7 RNAP transcripts, except for the *SOX7* RNA, every 200 ng contained more than 2.0 ng dsRNA, according to the dsRNA quantitative standard (Fig. 6C and 6D). However, dsRNA was barely detectable in the VSW-3 RNAP transcripts (Fig. 6C and 6D).

We tested whether the RdRp activity of T7 RNAP contributed to the production of full-length dsRNA. Using copGFP RNA produced by VSW-3 RNAP as the transcription template in an IVT reaction, no additional band was observed for either VSW-3 RNAP or T7 RNAP (Fig. 6E). Dot-blot analysis of these RNA products, VSW-3 (VSW-3) RNA and T7 (VSW-3) RNA, also showed that no dsRNA was detected (Fig. 6F). Although T7 (VSW-3) RNA products contained a 3’-loop-back dsRNA region shorter than 20 bp, the short loop-back dsRNA was not detected by J2 antibodies, as the J2 antibody only recognizes dsRNA larger than 40 bp [39]. These results confirmed that the full-length dsRNA byproducts in the IVT originated from the DNA template and not from the RNA template, consistent with a previous study [7].

**Figure 7.**
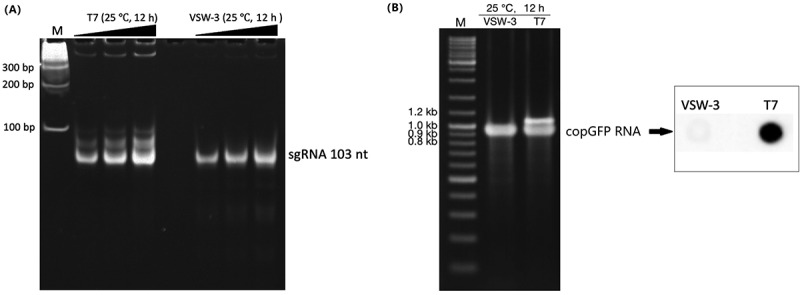
Comparison of the sgRNA (A) and copGFP RNA (B) synthesis by T7 or VSW-3 RNAP under the same IVT conditions (25°C for 12 h). The right panel in **(B)** shows the J2 antibody dot-blot detection of dsRNA (as in Fig. 6C) in the copGFP RNA products from the VSW-3 or T7 IVT. The terminal **(A)** and full-length dsRNA **(B)** by T7 RNAP were not reduced by lowering the IVT temperature.

IVT reaction conditions, such as Mg^2+^ and NTP concentrations, may affect full-length dsRNA production by T7 RNAP [5,7]. To verify whether the difference in full-length dsRNA production between T7 and VSW-3 RNAP was due to the difference in their optimal reaction temperature, we compared the synthesis of sgRNA (Fig. 7A) and copGFP RNA (Fig. 7B) by both enzymes using the same conditions (same IVT buffer, 25°C, for 12 h). The results confirmed that the terminal dsRNA (sgRNA, Fig. 7A) and the full-length dsRNA vivo(copGFP RNA, Fig. 7B) by T7 RNAP were not reduced by lowering the reaction temperature. Although reducing the Mg^2+^ and NTP concentration may further improve the RNA purity of the VSW-3 IVT, such as for T7 IVTs [5,7], the total dsRNA content in the VSW-3 IVT products was not significant using conditions for optimal yield.

The VSW-3 RNAP is the first ssRNAP characterized from psychrophilic phages. With a comparable RNA yield (but longer reaction time) to the routinely used T7 RNAP, the ability of VSW-3 RNAP to perform RNA synthesis at low temperatures may reduce RNA degradation during prolonged synthesis and in the absence of RNase inhibitors. In combination with its other advantages, including insensitivity to class II T7 transcription terminators, higher product 3’-terminal homogeneity, and importantly, minimal dsRNA production, the VSW-3 RNAP is especially advantageous for the synthesis of RNA for *in vivo* use, such as mRNA-based medicine. Indeed, previous studies have demonstrated superior cellular expression levels of mRNA (without modification) produced by VSW-3 RNAP [40][41].

## Supplementary Material

Supplemental Material

## Data Availability

The datasets generated during the current study are available from the corresponding author on reasonable request. Supplementary Data are available online.

## References

[1] Cheetham GM, Steitz TA. Insights into transcription: structure and function of single-subunit DNA-dependent RNA polymerases. Curr Opin Struct Biol. 2000;10(1):117–123. 10679468 10.1016/s0959-440x(99)00058-5

[2] Milligan JF, Groebe DR, Witherell GW, et al. Oligoribonucleotide synthesis using T7 RNA polymerase and synthetic DNA templates. Nucleic Acids Res. 1987;15:8783–8798. 3684574 10.1093/nar/15.21.8783 PMC306405

[3] Kropinski AM, Sulakvelidze A, Konczy P, et al. Salmonella phages and prophages–genomics and practical aspects. Methods Mol Biol. 2007;394:133–175. 18363236 10.1007/978-1-59745-512-1_9

[4] Wang W, Li Y, Wang Y, et al. Bacteriophage T7 transcription system: an enabling tool in synthetic biology. Biotechnol Adv. 2018;36:2129–2137. 30290194 10.1016/j.biotechadv.2018.10.001

[5] Wu MZ, Asahara H, Tzertzinis G, et al. Synthesis of low immunogenicity RNA with high-temperature in vitro transcription. RNA. 2020;26:345–360. 31900329 10.1261/rna.073858.119 PMC7025508

[6] Gholamalipour Y, Karunanayake Mudiyanselage A, Martin CT. 3’ end additions by T7 RNA polymerase are RNA self-templated, distributive and diverse in character-RNA-Seq analyses. Nucleic Acids Res. 2018;46:9253–9263. 30219859 10.1093/nar/gky796 PMC6182178

[7] Mu X, Greenwald E, Ahmad S, et al. An origin of the immunogenicity of in vitro transcribed RNA. Nucleic Acids Res. 2018;46:5239–5249. 29534222 10.1093/nar/gky177 PMC6007322

[8] Kariko K, Muramatsu H, Ludwig J, et al. Generating the optimal mRNA for therapy: HPLC purification eliminates immune activation and improves translation of nucleoside-modified, protein-encoding mRNA. Nucleic Acids Res. 2011;39:e142. 21890902 10.1093/nar/gkr695 PMC3241667

[9] Zhu B, Tabor S, Raytcheva DA, et al. The RNA polymerase of marine cyanophage Syn5. J Biol Chem. 2013;288:3545–3552. 23258537 10.1074/jbc.M112.442350 PMC3561573

[10] Lu X, Wu H, Xia H, et al. Klebsiella phage KP34 RNA polymerase and its use in RNA synthesis. Front Microbiol. 2019;10:2487. 31736920 10.3389/fmicb.2019.02487 PMC6834552

[11] Zhu B, Tabor S, Richardson CC. Syn5 RNA polymerase synthesizes precise run-off RNA products. Nucleic Acids Res. 2014;42(5):e33. 24285303 10.1093/nar/gkt1193 PMC3950665

[12] Zhu B, Hernandez A, Tan M, et al. Synthesis of 2’-Fluoro RNA by Syn5 RNA polymerase. Nucleic Acids Res. 2015;43:e94. 25897116 10.1093/nar/gkv367 PMC4538805

[13] Pollard C, De Koker S, Saelens X, et al. Challenges and advances towards the rational design of mRNA vaccines. Trends Mol Med. 2013;19:705–713. 24138818 10.1016/j.molmed.2013.09.002

[14] Sullenger BA, Nair S. From the RNA world to the clinic. Science. 2016;352:1417–1420. 27313039 10.1126/science.aad8709 PMC6035743

[15] Benteyn D, Heirman C, Bonehill A, et al. mRNA-based dendritic cell vaccines. Expert Rev Vaccines. 2015;14:161–176. 25196947 10.1586/14760584.2014.957684

[16] Zhang C, Zhang Z, Li J, et al. Complete genome sequence of the lytic cold-active *Pseudomonas fluorescens* bacteriophage VSW-3 from Napahai plateau wetland. Virus Genes. 2017;53:146–150. 27796639 10.1007/s11262-016-1403-1

[17] Qin K, Ji X, Zhang C, et al. Isolation and characterization of wetland VSW-3, a novel lytic cold-active bacteriophage of *Pseudomonas fluorescens*. Can J Microbiol. 2017;63:110–118. 28001438 10.1139/cjm-2016-0368

[18] Lyakhov DL, He B, Zhang X, et al. Mutant bacteriophage T7 RNA polymerases with altered termination properties. J Mol Biol. 1997;269:28–40. 9192998 10.1006/jmbi.1997.1015

[19] Lyakhov DL, He B, Zhang X, et al. Pausing and termination by bacteriophage T7 RNA polymerase. J Mol Biol. 1998;280:201–213. 9654445 10.1006/jmbi.1998.1854

[20] Macdonald LE, Durbin RK, Dunn JJ, et al. Characterization of two types of termination signal for bacteriophage T7 RNA polymerase. J Mol Biol. 1994;238:145–158. 8158645 10.1006/jmbi.1994.1277

[21] Pallan PS, Greene EM, Jicman PA, et al. Unexpected origins of the enhanced pairing affinity of 2’-fluoro-modified RNA. Nucleic Acids Res. 2011;39:3482–3495. 21183463 10.1093/nar/gkq1270 PMC3082899

[22] Pieken WA, Olsen DB, Benseler F, et al. Kinetic characterization of ribonuclease-resistant 2’-modified hammerhead ribozymes. Science. 1991;253:314–317. 1857967 10.1126/science.1857967

[23] Gibson DG, Young L, Chuang RY, et al. Enzymatic assembly of DNA molecules up to several hundred kilobases. Nat Methods. 2009;6:343–345. 19363495 10.1038/nmeth.1318

[24] Jain N, Blauch LR, Szymanski MR, et al. Transcription polymerase-catalyzed emergence of novel RNA replicons. Science. 2020;368:eaay0688. 32217750 10.1126/science.aay0688 PMC7445081

[25] Padilla R, Sousa R. Efficient synthesis of nucleic acids heavily modified with non-canonical ribose 2’-groups using a mutantT7 RNA polymerase (RNAP). Nucleic Acids Res. 1999;27:1561–1563. 10037823 10.1093/nar/27.6.1561 PMC148355

[26] Dunn JJ, Studier FW. Complete nucleotide sequence of bacteriophage T7 DNA and the locations of T7 genetic elements. J Mol Biol. 1983;166:477–535. 6864790 10.1016/s0022-2836(83)80282-4

[27] Martin CT, Coleman JE. Kinetic analysis of T7 RNA polymerase-promoter interactions with small synthetic promoters. Biochemistry. 1987;26:2690–2696. 3300768 10.1021/bi00384a006

[28] Krieg PA, Melton DA. In vitro RNA synthesis with SP6 RNA polymerase. Methods Enzymol. 1987;155:397–415. 2828872 10.1016/0076-6879(87)55027-3

[29] V V, Solovyev A,I, Shahmuradov. PromH: promoters identification using orthologous genomic sequences. Nucleic Acids Res. 2003;31:3540–3545. 12824362 10.1093/nar/gkg525 PMC168932

[30] Macdonald LE, Zhou Y, McAllister WT. Termination and slippage by bacteriophage T7 RNA polymerase. J Mol Biol. 1993;232:1030–1047. 8371265 10.1006/jmbi.1993.1458

[31] Milligan, John F. Synthesis of small RNAs using T7 RNA polymerase. Methods Enzymol. 1989;180:51–62. 2482430 10.1016/0076-6879(89)80091-6

[32] Cazenave C, Uhlenbeck OC. RNA template-directed RNA synthesis by T7 RNA polymerase. Proc Natl Acad Sci U S A. 1994;91:6972–6976. 7518923 10.1073/pnas.91.15.6972 PMC44320

[33] Triana-Alonso FJ, Dabrowski M, Wadzack J, et al. Self-coded 3’-extension of run-off transcripts produces aberrant products during in vitro transcription with T7 RNA polymerase. J Biol Chem. 1995;270:6298–6307. 7534310 10.1074/jbc.270.11.6298

[34] Gruber AR, Lorenz R, Bernhart SH, et al. The Vienna RNA websuite. Nucleic Acids Res. 2008;36:W70–74. 18424795 10.1093/nar/gkn188 PMC2447809

[35] Sousa R, Padilla R. A mutant T7 RNA polymerase as a DNA polymerase. EMBO J. 1995;14:4609–4621. 7556104 10.1002/j.1460-2075.1995.tb00140.x PMC394553

[36] Ali S, Jesse G, Inamati GB, et al. Hybridization of 2′-ribose modified mixed-sequence oligonucleotides: thermodynamic and kinetic studies. Nucleic Acids Res. 2001;29:2163–2170. 11353086 10.1093/nar/29.10.2163 PMC55455

[37] Ono T, Scalf M, Smith LM. 2’-Fluoro modified nucleic acids: polymerase-directed synthesis, properties and stability to analysis by matrix-assisted laser desorption/ionization mass spectrometry. Nucleic Acids Res. 1997;25:4581–4588. 9358169 10.1093/nar/25.22.4581 PMC147098

[38] Poynter SJ, DeWitte-Orr SJ. Understanding viral dsRNA-mediated innate immune responses at the cellular level using a rainbow trout model. Front Immunol. 2018;9:829. 29740439 10.3389/fimmu.2018.00829 PMC5924774

[39] Schonborn J, Oberstraβ J, Breyel E, et al. Monoclonal antibodies to double-stranded RNA as probes of RNA structure in crude nucleic acid extracts. Nucleic Acids Res. 1991;19:2993–3000. 2057357 10.1093/nar/19.11.2993 PMC328262

[40] Ma X, Tan X, Yu B, et al. DOCK2 regulates antifungal immunity by regulating RAC GTPase activity. Cell Mol Immunol. 2022;19:602–618. 35079145 10.1038/s41423-021-00835-0 PMC8787451

[41] Wang G, Cheng R, Chen Q, Xu Y, Yu B, Zhu B, Yin H and Xia H. (2022). mRNA produced by VSW-3 RNAP has high-level translation efficiency with low inflammatory stimulation. Cell Insight, 1(5), 100056 10.1016/j.cellin.2022.100056 PMC10120321 37193555

