# Supplementary material for: Psychrophilic phage VSW-3 RNA polymerase reduces both terminal and full-length dsRNA byproducts in in vitro transcription
Source: RNA Biol. 2022 Oct 26;19(1):1130–42. doi: 10.1080/15476286.2022.2139113 (PMC9624206; doi:10.1080/15476286.2022.2139113)
Supplement: Supplemental Material [file KRNB_A_2139113_SM0997.pdf]

Supplementary Materials

Table S1. Nucleotide sequences of VSW-3 promoter and VSW-3 RNAP (and Y578F mutant).

**VSW-3 promoter:**	TTAATTGGGCCACCTATA

Nucleotide sequences of VSW-3 RNAP (WT):

ATGAACCAGATCGAGCTAGAACAGGAAATGATTGACGGTGGCCGGGCGAAGATGTTCGGCTCATTCAATCGCAACGAAGAGCAA
GGAGCGGCGCACAACAACCCATACGCCGCAGCGGTGTACCGGCGATTCGTGCAACCTCTGGCCGATCAAATCGACGCCTACTG
CGGTGAGGTCAAGCGCGGCGTGATGGCGGCAGGCAAAGCCCTGCTGCGCCCGCATGACCCGATGGTGTTGGCGTTCATGACCG
TTCGCATGGTCATGGACACCACGCTGCAATCGAAGGACAACGCACCAACCGCTGTGGCCCGAGCCTTGGGCCAGAGCATCTACG
GGGAGACTCTGCTCGCCAAGTTTGAGCAGGTCGAACCCGACCTATACTTCACGCTGGTCAATGACTTTGAGCGGCGTATGACCAA
GTCGGAGCGGCACCGGCTGACGGTTTTCAAGATGCAGGCCGAGAAGAACGGCGTACCGCTGCCTGTGTGGTCGCCAGAGGACA
AGTTGGCCATCGGCACTATCTTGCTCTACCTTGCCCGCGATGTCGGGCTGGTGGAGATCACAGAGGTGCGCAAGGGCAAGAAGA
CTGTGCGCGAGTACAACATGACGCCGGATGTGGCGGGCATGCTTGACAACATCAAGGACTTTGTGGCAGGGGCCAGCCCGATG
GTGCTGCCTTGTGTGGTGCCTCCGGTGCCATGGACTGATGCCAACAACGGAGGATACCACACACCGGGCATGCGCCGCATAAGC
CCCTGCTGCATCCGTGGGCGACCGCGAGTCGAAGACCTGACCGATGTACCGGACATCCCGTTGCGTGCGCTCAACATCCTCCAG
AGCCGCCCATGGCGCATCAATCGCATGGTGTTGGACGCGGTGGATCTGGTGGGCCAGCGGTTCGACGTGGGTGAGGTGCTGGC
ACAGGCCGAGCTGCCGAAGCCGAAGTCGCTTCTGTGGCTGGACGATGTGCCGAAGGAAGAAATGAACCCCGCGCAACTGGCCG
AGTTCGGTGCGTGGAAGATCGAGATGCGCGAGTGGTACACCGAGAACAAGAGCAGGGGCGTGCAGTGGGGCCGGTACTATGAG
GCGCTGCGAGTAGCCCGCAAGTTCAAGGACTTGCCGTTCTGGTTCGTGTACCAATACGACTACCGAGGCCGAGCATATGCGAAC
ACGAGGGGCGTTAGCCCGCAAGGTTCAGATCTCCAGAAGGCGCTGCTTATGGCAGACGTTGGCGTCCCAATCGCCGACGAACG
AGCCAAGTTCTGGTTCTACACAGCCGGAGCAAACCGGTTCGGGTACGACAAAGCCACACTGGCAGAGAGGTACGAATGGACTGT
AGAACGCTCGGAAATGATCTGTGCTATTGCTGCCGATCCCGTAGCCAACAGGCAATGGACGGAGGCGGACAACCCGTTCCAGTT
TCTCGCATGGTGCTTCGAGTTCGCCCAGTACACGGCAATGCCCGAGAGCTTCTTATCTCGCCTCGCTCTTGGACAGGATGGGAG
CTGCAACGGGCTACAGCACTTCTCAGCGATGTTGCGCGACGAAGTGGGTGGACTCGCGACCAACTTAGTGCCCTCTACAACGCA
GCAGGACATCTATCGACTGGTAGCTGTGGAGACAACGCGGTTGTTACAAGCTATGCCTCACGAGAACTGCGAGTTCACGCTGAA
GTGGAAGCTGCACAGCCTGTCCCGCGACTTAGTCAAACGAAGCGTTATGACTTTGCCGTATGGATCGACGAGGTTCAGTTGTGCT
GACTTCATCTACACCGAGTACATGGCGAAGCACAAGGCGCCGGAGTTCGCCAAGGGCGACTACCAGAAGGCCGCTCGCTGGCT
GAGCGTACCGGTGTGGGACGCAATCGGCAACGTAGTGGTCAAGGCAAGAGAGGCGATGGCATGGCTTCAGAACGCCTCTGACG
AGCTGATAGACGCCGGGATCGACGAGATCTACTGGCGGTCGCCAAGCGGATTCATGGTTCGGCAACGGTACGGCAAGGAAGAAT
TCGTTCTTGTCAAGACTCGATTGGCTGGCGGAGTCAGAATTCGGCCAACCATCAAGCTGGAGCTAGAGGAACCATGCAAGCGCC
GGCACCGGAACGGGATAGCTCCCAACTTCGTTCACAGCCACGACGCCGCGCACATGCACCTCCTGATCTGCGCCGCCGAGGAT
CATGGGCTGGGCCATCTGGCATTCATCCATGACGACTACGGTACGACTGCGGATGGTACTGAAACGCTCCACAAGCTCATCAGG
GCGACGTTCGTTGCCATGTACGAGCAAGGGTGCCCATTGACCGCATTCCGCGACACATACGGCATCACAGAAGATCTCCCGGAA
CGCGGTGATCTCGACCTGAATCTGGTTCACGATTCCACGTATTTCTTCGCCTGA

Nucleotide sequences of VSW-3 RNAP Y578F:

ATGAACCAGATCGAGCTAGAACAGGAAATGATTGACGGTGGCCGGGCGAAGATGTTCGGCTCATTCAATCGCAACGAAGAGCAA
GGAGCGGCGCACAACAACCCATACGCCGCAGCGGTGTACCGGCGATTCGTGCAACCTCTGGCCGATCAAATCGACGCCTACTG
CGGTGAGGTCAAGCGCGGCGTGATGGCGGCAGGCAAAGCCCTGCTGCGCCCGCATGACCCGATGGTGTTGGCGTTCATGACCG
TTCGCATGGTCATGGACACCACGCTGCAATCGAAGGACAACGCACCAACCGCTGTGGCCCGAGCCTTGGGCCAGAGCATCTACG
GGGAGACTCTGCTCGCCAAGTTTGAGCAGGTCGAACCCGACCTATACTTCACGCTGGTCAATGACTTTGAGCGGCGTATGACCAA
GTCGGAGCGGCACCGGCTGACGGTTTTCAAGATGCAGGCCGAGAAGAACGGCGTACCGCTGCCTGTGTGGTCGCCAGAGGACA
AGTTGGCCATCGGCACTATCTTGCTCTACCTTGCCCGCGATGTCGGGCTGGTGGAGATCACAGAGGTGCGCAAGGGCAAGAAGA
CTGTGCGCGAGTACAACATGACGCCGGATGTGGCGGGCATGCTTGACAACATCAAGGACTTTGTGGCAGGGGCCAGCCCGATG
GTGCTGCCTTGTGTGGTGCCTCCGGTGCCATGGACTGATGCCAACAACGGAGGATACCACACACCGGGCATGCGCCGCATAAGC
CCCTGCTGCATCCGTGGGCGACCGCGAGTCGAAGACCTGACCGATGTACCGGACATCCCGTTGCGTGCGCTCAACATCCTCCAG
AGCCGCCCATGGCGCATCAATCGCATGGTGTTGGACGCGGTGGATCTGGTGGGCCAGCGGTTCGACGTGGGTGAGGTGCTGGC
ACAGGCCGAGCTGCCGAAGCCGAAGTCGCTTCTGTGGCTGGACGATGTGCCGAAGGAAGAAATGAACCCCGCGCAACTGGCCG
AGTTCGGTGCGTGGAAGATCGAGATGCGCGAGTGGTACACCGAGAACAAGAGCAGGGGCGTGCAGTGGGGCCGGTACTATGAG
GCGCTGCGAGTAGCCCGCAAGTTCAAGGACTTGCCGTTCTGGTTCGTGTACCAATACGACTACCGAGGCCGAGCATATGCGAAC
ACGAGGGGCGTTAGCCCGCAAGGTTCAGATCTCCAGAAGGCGCTGCTTATGGCAGACGTTGGCGTCCCAATCGCCGACGAACG
AGCCAAGTTCTGGTTCTACACAGCCGGAGCAAACCGGTTCGGGTACGACAAAGCCACACTGGCAGAGAGGTACGAATGGACTGT
AGAACGCTCGGAAATGATCTGTGCTATTGCTGCCGATCCCGTAGCCAACAGGCAATGGACGGAGGCGGACAACCCGTTCCAGTT
TCTCGCATGGTGCTTCGAGTTCGCCCAGTACACGGCAATGCCCGAGAGCTTCTTATCTCGCCTCGCTCTTGGACAGGATGGGAG
CTGCAACGGGCTACAGCACTTCTCAGCGATGTTGCGCGACGAAGTGGGTGGACTCGCGACCAACTTAGTGCCCTCTACAACGCA
GCAGGACATCTATCGACTGGTAGCTGTGGAGACAACGCGGTTGTTACAAGCTATGCCTCACGAGAACTGCGAGTTCACGCTGAA
GTGGAAGCTGCACAGCCTGTCCCGCGACTTAGTCAAACGAAGCGTTATGACTTTGCCGTTTGGATCGACGAGGTTCAGTTGTGCT
GACTTCATCTACACCGAGTACATGGCGAAGCACAAGGCGCCGGAGTTCGCCAAGGGCGACTACCAGAAGGCCGCTCGCTGGCT
GAGCGTACCGGTGTGGGACGCAATCGGCAACGTAGTGGTCAAGGCAAGAGAGGCGATGGCATGGCTTCAGAACGCCTCTGACG
AGCTGATAGACGCCGGGATCGACGAGATCTACTGGCGGTCGCCAAGCGGATTCATGGTTCGGCAACGGTACGGCAAGGAAGAAT
TCGTTCTTGTCAAGACTCGATTGGCTGGCGGAGTCAGAATTCGGCCAACCATCAAGCTGGAGCTAGAGGAACCATGCAAGCGCC
GGCACCGGAACGGGATAGCTCCCAACTTCGTTCACAGCCACGACGCCGCGCACATGCACCTCCTGATCTGCGCCGCCGAGGAT
CATGGGCTGGGCCATCTGGCATTCATCCATGACGACTACGGTACGACTGCGGATGGTACTGAAACGCTCCACAAGCTCATCAGG
GCGACGTTCGTTGCCATGTACGAGCAAGGGTGCCCATTGACCGCATTCCGCGACACATACGGCATCACAGAAGATCTCCCGGAA
CGCGGTGATCTCGACCTGAATCTGGTTCACGATTCCACGTATTTCTTCGCCTGA

Table S2. DNA oligos for the preparation of IVT templates.

**Primers for PCR amplification of the cas9 RNA IVT template**	
**Trans_Template-cas9-F :**	AGCTGGTTTAGTGAACCGTCAGATC
**Trans_Template-cas9-R :**	ACTCAATGGTGATGGTGATGATGACC
**DNA oligos for the construction of DNA templates containing truncated VSW-3 RNAP promoters**	
**VSW3-promoter Test (19)-F:**	**TTTAATTGGGCCACCTATA**GTACACGGGCAGCTTGCCGGGTTTTAGAGCTAGAAATAGC
**VSW3-promoter Test (19)-R:**	GCTATTTCTAGCTCTAAAACCCGGCAAGCTGCCCGTGTAC**TATAGGTGGCCCAATTAAA**
**VSW3-promoter Test (18)-F:**	**TTAATTGGGCCACCTATA**GTACACGGGCAGCTTGCCGGGTTTTAGAGCTAGAAATAGC
**VSW3-promoter Test (18)-R:**	GCTATTTCTAGCTCTAAAACCCGGCAAGCTGCCCGTGTAC**TATAGGTGGCCCAATTAA**
**VSW3-promoter Test (17)-F:**	**TAATTGGGCCACCTATA**GTACACGGGCAGCTTGCCGGGTTTTAGAGCTAGAAATAGC
**VSW3-promoter Test (17)-R:**	GCTATTTCTAGCTCTAAAACCCGGCAAGCTGCCCGTGTAC**TATAGGTGGCCCAATTA**
**VSW3-promoter Test (16)-F:**	**AATTGGGCCACCTATA**GTACACGGGCAGCTTGCCGGGTTTTAGAGCTAGAAATAGC
**VSW3-promoter Test (16)-R:**	GCTATTTCTAGCTCTAAAACCCGGCAAGCTGCCCGTGTAC**TATAGGTGGCCCAATT**
**VSW3-promoter Test (15)-F:**	**ATTGGGCCACCTATA**GTACACGGGCAGCTTGCCGGGTTTTAGAGCTAGAAATAGC
**VSW3-promoter Test (15)-R:**	GCTATTTCTAGCTCTAAAACCCGGCAAGCTGCCCGTGTAC**TATAGGTGGCCCAAT**
**VSW3-promoter Test (14)-F:**	**TTGGGCCACCTATA**GTACACGGGCAGCTTGCCGGGTTTTAGAGCTAGAAATAGC
**VSW3-promoter Test (14)-R:**	GCTATTTCTAGCTCTAAAACCCGGCAAGCTGCCCGTGTAC**TATAGGTGGCCCAA**

Table S3. Sequences (5’-3’) of pUC19-, sox7, tdTomato, copGFP, cas9 and eGFP RNA.

**pUC19-RNA sequence (sequence in red indicates the class I T7 terminator)**
GGGUCUAGAGUCGACCUGCAGGCAUGCAAGCUUGGCGUAAUCAUGGUCAUAGCUGUUUCCUGUGUGAAAUUGUUAUCCGC
UCACAAUUCCACACAACAUACGAGCCGGAAGCAUAAAGUGUAAAGCCUGGGGUGCCUAAUGAGUGAGCUAACUCACAUUAAU
UGCGUUGCGCUCACUGCCCGCUUUCCAGUCGGGAAACCUGUCGUGCCAGCUGCAUUAAUGAAUCGGCCAACGCGCGGGGA
GAGGCGGUUUGCGUAUUGGGCGCUCUUCCGCUUCCUCGCUCACUGACUCGCUGCGCUCGGUCGUUCGGCUGCGGCGAGC
GGUAUCAGCUCACUCAAAGGCGGUAAUACGGUUAUCCACAGAAUCAGGGGAUAACGCAGGAAAGAACAUGUGAGCAAAAGG
CCAGCAAAAGGCCAGGAACCGUAAAAAGGCCGCGUUGCUGGCGUUUUUCCAUAGGCUCCGCCCCCCUGACGAGCAUCACAA
AAAUCGACGCUCAAGUCAGAGGUGGCGAAACCCGACAGGACUAUAAAGAUACCAGGCGUUUCCCCCUGGAAGCUCCCUCGU
GCGCUCUCCUGUUCCGACCCUGCCGCUUACCGGAUACCUGUCCGCCUUUCUCCCUUCGGGAAGCGUGGCGCUUUCUCAUA
GCUCACGCUGUAGGUAUCUCAGUUCGGUGUAGGUCGUUCGCUCCAAGCUGGGCUGUGUGCACGAACCCCCCGUUCAGCCC
GACCGCUGCGCCUUAUCCGGUAACUAUCGUCUUGAGUCCAACCCGGUAAGACACGACUUAUCGCCACUGGCAGCAGCCACU
GGUAACAGGAUUAGCAGAGCGAGGUAUGUAGGCGGUGCUACAGAGUUCUUGAAGUGGUGGCCUAACUACGGCUACACUAG
AAGAACAGUAUUUGGUAUCUGCGCUCUGCUGAAGCCAGUUACCUUCGGAAAAAGAGUUGGUAGCUCUUGAUCCGGCAAACA
AACCACCGCUGGUAGCGGUGGUUUUUUUGUUUGCAAGCAGCAGAUUACGCGCAGAAAAAAAGGAUCUCAAGAAGAUCCUUU
GAUCUUUUCUACGGGGUCUGACGCUCAGUGGAACGAAAACUCACGUUAAGGGAUUUUGGUCAUGAGAUUAUCAAAAAGGAU
CUUCACCUAGAUCCUUUUAAAUUAAAAAUGAAGUUUUAAAUCAAUCUAAAGUAUAUAUGAGUAAACUUGGUCUGACAGUUAC
CAAUGCUUAAUCAGUGAGGCACCUAUCUCAGCGAUCUGUCUAUUUCGUUCAUCCAUAGUUGCCUGACUCCCCGUCGUGUAG
AUAACUACGAUACGGGAGGGCUUACCAUCUGGCCCCAGUGCUGCAAUGAUACCGCGAGACCCACGCUCACCGGCUCCAGAU
UUAUCAGCAAUAAACCAGCCAGCCGGAAGGGCCGAGCGCAGAAGUGGUCCUGCAACUUUAUCCGCCUCCAUCCAGUCUAUU
AAUUGUUGCCGGGAAGCUAGAGUAAGUAGUUCGCCAGUUAAUAGUUUGCGCAACGUUGUUGCCAUUGCUACAGGCAUCGU
GGUGUCACGCUCGUCGUUUGGUAUGGCUUCAUUCAGCUCCGGUUCCCAACGAUCAAGGCGAGUUACAUGAUCCCCCAUGU
UGUGCAAAAAAGCGGUUAGCUCCUUCGGUCCUCCGAUCGUUGUCAGAAGUAAGUUGGCCGCAGUGUUAUCACUCAUGGUU
AUGGCAGCACUGCAUAAUUCUCUUACUGUCAUGCCAUCCGUAAGAUGCUUUUCUGUGACUGGUGAGUACUCAACCAAGUCA
UUCUGAGAAUAGUGUAUGCGGCGACCGAGUUGCUCUUGCCCGGCGUCAAUACGGGAUAAUACCGCGCCACAUAGCAGAACU
UUAAAAGUGCUCAUCAUUGGAAAACGUUCUUCGGGGCGAAAACUCUCAAGGAUCUUACCGCUGUUGAGAUCCAGUUCGAUG
UAACCCACUCGUGCACCCAACUGAUCUUCAGCAUCUUUUACUUUCACCAGCGUUUCUGGGUGAGCAAAAACAGGAAGGCAA
AAUGCCGCAAAAAAGGGAAUAAGGGCGACACGGAAAUGUUGAAUACUCAUACUCUUCCUUUUUCAAUAUUAUUGAAGCAUUU
AUCAGGGUUAUUGUCUCAUGAGCGGAUACAUAUUUGAAUGUAUUUAGAAAAAUAAACAAAUAGGGGUUCCGCGCACAUUUC
CCCGAAAAGUGCCACCUGACGUCUAAGAAACCAUUAUUAUCAUGACAUUAACCUAUAAAAAUAGGCGUAUCACGAGGCCCUU
UCGUCUCGCGCGUUUCGGUGAUGACGGUGAAAACCUCUGACACAUGCAGCUCCCGGAGACGGUCACAGCUUGUCUGUAAG
CGGAUGCCGGGAGCAGACAAGCCCGUCAGGGCGCGUCAGCGGGUGUUGGCGGGUGUCGGGGCUGGCUUAACUAUGCGGC
AUCAGAGCAGAUUGUACUGAGAGUGCACCAUA
**sox7 RNA sequence (GenBank: NM_031439.4)**
GGGAGACCCUCGAGGACAGAUCGCCUGGAGACGGCAAGAGCCGCCACCAUGAAAAGGCCGGCGGCCACGAAAAAGGCCGG
CCAGGCAAAAAAGAAAAAGGGUUCUGGAGCUUCGCUGCUGGGAGCCUACCCUUGGCCCGAGGGUCUCGAGUGCCCGGCCC
UGGACGCCGAGCUGUCGGAUGGACAAUCGCCGCCGGCCGUCCCCCGGCCCCCGGGGGACAAGGGCUCCGAGAGCCGUAU
CCGGCGGCCCAUGAACGCCUUCAUGGUUUGGGCCAAGGACGAGAGGAAACGGCUGGCAGUGCAGAACCCGGACCUGCACA
ACGCCGAGCUCAGCAAGAUGCUGGGAAAGUCGUGGAAGGCGCUGACGCUGUCCCAGAAGAGGCCGUACGUGGACGAGGCG
GAGCGGCUGCGCCUGCAGCACAUGCAGGACUACCCCAACUACAAGUACCGGCCGCGCAGGAAGAAGCAGGCCAAGCGGCU
GUGCAAGCGCGUGGACCCGGGCUUCCUUCUGAGCUCCCUCUCCCGGGACCAGAACGCCCUGCCGGAGAAGAGAAGCGGCA
GCCGGGGGGCGCUGGGGGAGAAGGAGGACAGGGGUGAGUACUCCCCCGGCACUGCCCUGCCCAGCCUCCGGGGCUGCUA
CCACGAGGGGCCGGCUGGUGGUGGCGGCGGCGGCACCCCGAGCAGUGUGGACACGUACCCGUACGGGCUGCCCACACCU
CCUGAAAUGUCUCCCCUGGACGUGCUGGAGCCGGAGCAGACCUUCUUCUCCUCCCCCUGCCAGGAGGAGCAUGGCCAUCC
CCGCCGCAUCCCCCACCUGCCAGGGCACCCGUACUCACCGGAGUACGCCCCAAGCCCUCUCCACUGUAGCCACCCCCUGG
GCUCCCUGGCCCUUGGCCAGUCCCCCGGCGUCUCCAUGAUGUCCCCUGUACCCGGCUGUCCCCCAUCUCCUGCCUAUUAC
UCCCCGGCCACCUACCACCCACUCCACUCCAACCUCCAAGCCCACCUGGGCCAGCUUUCCCCGCCUCCUGAGCACCCUGGC
UUCGACGCCCUGGAUCAACUGAGCCAGGUGGAACUCCUGGGGGACAUGGAUCGCAAUGAAUUCGACCAGUAUUUGAACACU
CCUGGCCACCCAGACUCCGCCACAGGGGCCAUGGCCCUCAGUGGGCAUGUUCCGGUCUCCCAGGUGACACCAACGGGUCC
CACAGAGACCAGCCUCAUCUCCGUCCUGGCUGAUGCCACGGCCACGUACUACAACAGCUACAGUGUGUCAGGAUCCCCCAA
GAAGAAGAGGAAAGUCUCGAGCGACUACAAAGACCAUGACGGUGAUUAUAAAGAUCAUGACAUCGAUUACAAGGAUGACGAU
GACAAGGCUGCAGGAUGAA
**tdTomato RNA sequence (GenBank: KT878736.1)**
GGGAGACCCUCGAGGACAGAUCGCCUGGAGACGCCAUCCACGCUGUUUUGACCUCCAUAGAAGACACCGGGACCGAUCCA
GCCUCCGCGGCCGGGAACGGUGCAUUGGAACGCGGAUUCCCCGUGCCAAGAGUGACUCACCGUCCUUGACACGACACGAU
GAUAAUAUGGUGAGCAAGGGCGAGGAGGUCAUCAAAGAGUUCAUGCGCUUCAAGGUGCGCAUGGAGGGCUCCAUGAACGG
CCACGAGUUCGAGAUCGAGGGCGAGGGCGAGGGCCGCCCCUACGAGGGCACCCAGACCGCCAAGCUGAAGGUGACCAAGG
GCGGCCCCCUGCCCUUCGCCUGGGACAUCCUGUCCCCCCAGUUCAUGUACGGCUCCAAGGCGUACGUGAAGCACCCCGCC
GACAUCCCCGAUUACAAGAAGCUGUCCUUCCCCGAGGGCUUCAAGUGGGAGCGCGUGAUGAACUUCGAGGACGGCGGUCU
GGUGACCGUGACCCAGGACUCCUCCCUGCAGGACGGCACGCUGAUCUACAAGGUGAAGAUGCGCGGCACCAACUUCCCCC
CCGACGGCCCCGUAAUGCAGAAGAAGACCAUGGGCUGGGAGGCCUCCACCGAGCGCCUGUACCCCCGCGACGGCGUGCUG
AAGGGCGAGAUCCACCAGGCCCUGAAGCUGAAGGACGGCGGCCACUACCUGGUGGAGUUCAAGACCAUCUACAUGGCCAA
GAAGCCCGUGCAACUGCCCGGCUACUACUACGUGGACACCAAGCUGGACAUCACCUCCCACAACGAGGACUACACCAUCGU
GGAACAGUACGAGCGCUCCGAGGGCCGCCACCACCUGUUCCUGGGGCAUGGCACCGGCAGCACCGGCAGCGGCAGCUCCG
GCACCGCCUCCUCCGAGGACAACAACAUGGCCGUCAUCAAAGAGUUCAUGCGCUUCAAGGUGCGCAUGGAGGGCUCCAUGA
ACGGCCACGAGUUCGAGAUCGAGGGCGAGGGCGAGGGCCGCCCCUACGAGGGCACCCAGACCGCCAAGCUGAAGGUGACC
AAGGGCGGCCCCCUGCCCUUCGCCUGGGACAUCCUGUCCCCCCAGUUCAUGUACGGCUCCAAGGCGUACGUGAAGCACCC
CGCCGACAUCCCCGAUUACAAGAAGCUGUCCUUCCCCGAGGGCUUCAAGUGGGAGCGCGUGAUGAACUUCGAGGACGGCG
GUCUGGUGACCGUGACCCAGGACUCCUCCCUGCAGGACGGCACGCUGAUCUACAAGGUGAAGAUGCGCGGCACCAACUUC
CCCCCCGACGGCCCCGUAAUGCAGAAGAAGACCAUGGGCUGGGAGGCCUCCACCGAGCGCCUGUACCCCCGCGACGGCGU
GCUGAAGGGCGAGAUCCACCAGGCCCUGAAGCUGAAGGACGGCGGCCACUACCUGGUGGAGUUCAAGACCAUCUACAUGG
CCAAGAAGCCCGUGCAACUGCCCGGCUACUACUACGUGGACACCAAGCUGGACAUCACCUCCCACAACGAGGACUACACCA
UCGUGGAACAGUACGAGCGCUCCGAGGGCCGCCACCACCUGUUCCUGUACGGCAUGGACGAGCUGUACAAGUGAACGCGU
CUGGAACAAUCGGGUGGCAUCCCUGUGACCCCUCCCCAGUGCCUCUCCUGGCCCUGGAAGUUGCCACUCCAGUGCCCACC
AGCCUUGUCCUAAUAAAAUUAAGUUGCAUCAAGCUCUA
**copGFP RNA sequence (GenBank: KX757255.1)**
GGGAGAGCCGCCACCAUGGAGAGCGACGAGAGCGGCCUGCCCGCCAUGGAGAUCGAGUGCCGCAUCACCGGCACCCUGAA
CGGCGUGGAGUUCGAGCUGGUGGGCGGCGGAGAGGGCACCCCCAAGCAGGGCCGCAUGACCAACAAGAUGAAGAGCACCA

AAGGCGCCCUGACCUUCAGCCCCUACCUGCUGAGCCACGUGAUGGGCUACGGCUUCUACCACUUCGGCACCUACCCCAGC
GGCUACGAGAACCCCUUCCUGCACGCCAUCAACAACGGCGGCUACACCAACACCCGCAUCGAGAAGUACGAGGACGGCGGC
GUGCUGCACGUGAGCUUCAGCUACCGCUACGAGGCCGGCCGCGUGAUCGGCGACUUCAAGGUGGUGGGCACCGGCUUCC
CCGAGGACAGCGUGAUCUUCACCGACAAGAUCAUCCGCAGCAACGCCACCGUGGAGCACCUGCACCCCAUGGGCGAUAACG
UGCUGGUGGGCAGCUUCGCCCGCACCUUCAGCCUGCGCGACGGCGGCUACUACAGCUUCGUGGUGGACAGCCACAUGCAC
UUCAAGAGCGCCAUCCACCCCAGCAUCCUGCAGAACGGGGGCCCCAUGUUCGCCUUCCGCCGCGUGGAGGAGCUGCACAG
CAACACCGAGCUGGGCAUCGUGGAGUACCAGCACGCCUUCAAGACCCCCAUCGCCUUCGCCAGAUCCCGCGCUCAGUCGU
CCAAUUCUGCCGUGGACGGCACCGCCGGACCCGGCUCCACCGGAUCUCGCUCCCCCAAGAAGAAGAGGAAAGUCUCGAGC
GACUACAAAGACCUAUAGGUGGCCCAAUUAAUGACGGUGAUUAUAAAGAUCAUGACAUCGAUUACAAGGAUGACGAUGACAA
GGCGCAGGAUGACCGGUCAUCAUCACCAUCACCAUUGAGU

copGFP RNA complementary sequence (-)

GGUCUUUGUAGUCGCUCGAGACUUUCCUCUUCUUCUUGGGGCUAGCCUAGCGAGAUCCGGUGGAGCCGGGUCCGGCGGU
GCCGUCCACGGCAGAAUUGGACGACUGAGCGCGGAUCUGGCGAAGGCGAUGGGGGUCUUGAAGGCGUGCUGGUACUCC
ACGAUGCCCAGCUCGGUGUUGCUGUGCAGCUCCUCCACGCGGCGGAAGGCGAACAUGGGGCCCCCGUUCUGCAGGAUGCU
GGGGUGGAUGGCGCUCUUGAAGUGCAUGUGGCUGUCCACCACGAAGCUGUAGUAGCCGCCGUCGCGCAGGCUGAAGGUG
CGGGCGAAGCUGCCCACCAGCACGUUAUCGCCCAUGGGGUGCAGGUGCUCCACGGUGGCGUUGCUGCGGAUGAUCUUGU
CGGUGAAGAUCACGCUGUCCUCGGGGAAGCCGGUGCCCACCACCUUGAAGUCGCCGAUCACGCGGCCGGCCUCGUAGCGG
UAGCUGAAGCUCACGUGCAGCACGCCGCCGUCCUCGUACUUCUCGAUGCGGGUGUUGGUGUAGCCGCCGUUGUUGAUGG
CGUGCAGGAAGGGGUUCUCGUAGCCGCUGGGGUAGGUGCCGAAGUGGUAGAAGCCGUAGCCCAUCACGUGGCUCAGCAG
GUAGGGGCUGAAGGUCAGGGCGCCUUUGGUGCUCUUCAUCUUGUUGGUCAUGCGGCCCUGCUUGGGGGUGCCCUCUCCG
CCGCCCACCAGCUCGAACUCCACGCCGUUCAGGGUGCCGGUGAUGCGGCACUCGAUCUCCAUGGCGGGCAGGCCGCUCUC
GUCGCUCUCCAUGGUGGCGGCUCUC

cas9 RNA sequence (Addgene: 72247)

GGGAGAGCCGCCACCAUGGAUAAAAAGUAUUCUAUUGGUUUAGACAUCGGCACUAAUUCCGUUGGAUGGGCUGUCAUAACC
GAUGAAUACAAAGUACCUUCAAAGAAAUUUAAGGUGUUGGGGAACACAGACCGUCAUUCGAUUAAAAAGAAUCUUAUCGGUG
CCCUCCUAUUCGAUAGUGGCGAAACGGCAGAGGCGACUCGCCUGAAACGAACCGCUCGGAGAAGGUAUACACGUCGCAAGA
ACCGAAUAUGUUACUUACAAGAAAUUUUUAGCAAUGAGAUGGCCAAAGUUGACGAUUCUUUCUUUCACCGUUUGGAAGAGU
CCUUCCUUGUCGAAGAGGACAAGAAACAUGAACGGCACCCCAUCUUUGGAAACAUAGUAGAUGAGGUGGCAUAUCAUGAAA
AGUACCCAACGAUUUAUCACCUCAGAAAAAAGCUAGUUGACUCAACUGAUAAAGCGGACCUGAGGUUAAUCUACUUGGCUCU
UGCCCAUAUGAUAAAGUUCCGUGGGCACUUUCUCAUUGAGGGUGAUCUAAAUCCGGACAACUCGGAUGUCGACAAACUGUU
CAUCCAGUUAGUACAAACCUAUAAUCAGUUGUUUGAAGAGAACCCUAUAAAUGCAAGUGGCGUGGAUGCGAAGGCUAUUCU
UAGCGCCCGCCUCUCUAAAUCCCGACGGCUAGAAAACCUGAUCGCACAAUUACCCGGAGAGAAGAAAAAUGGGUUGUUCGG
UAACCUUAUAGCGCUCUCACUAGGCCUGACACCAAAUUUUAAGUCGAACUUCGACUUAGCUGAAGAUGCCAAAUUGCAGCU
UAGUAAGGACACGUACGAUGACGAUCUCGACAAUCUACUGGCACAAAUUGGAGAUCAGUAUGCGGACUUAUUUUUGGCUGC
CAAAAACCUUAGCGAUGCAAUCCUCCUAUCUGACAUACUGAGAGUUAAUACUGAGAUUACCAAGGCGCCGUUAUCCGCUUCA
AUGAUCAAAAGGUACGAUGAACAUCACCAAGACUUGACACUUCUCAAGGCCCUAGUCCGUCAGCAACUGCCUGAGAAAUAUA
AGGAAAUAUUCUUUGAUCAGUCGAAAAACGGGUACGCAGGUUAUAUUGACGGCGGAGCGAGUCAAGAGGAAUUCUACAAGU
UUAUCAAACCCAUAUUAGAGAAGAUGGAUGGGACGGAAGAGUUGCUUGUAAAACUCAAUCGCGAAGAUCUACUGCGAAAGC
AGCGGACUUUCGACAACGGUAGCAUUCCACAUCAAAUCCACUUAGGCGAAUUGCAUGCUAUACUUAGAAGGCAGGAGGAUU
UUUAUCCGUUCCUCAAAGACAAUCGUGAAAAGAUUGAGAAAAUCCUAACCUUUCGCAUACCUUACUAUGUGGGACCCCUGG
CCCGAGGGAACUCUCGGUUCGCAUGGAUGACAAGAAAGUCCGAAGAAACGAUUACUCCCUGGAAUUUUGAGGAAGUUGUCG
AUAAAGGUGCGUCAGCUCAAUCGUUCAUCGAGAGGAUGACCGCCUUUGACAAGAAUUUACCGAACGAAAAAGUAUUGCCUA
AGCACAGUUUACUUUACGAGUAUUUCACAGUGUACAAUGAACUCACGAAAGUUAAGUAUGUCACUGAGGGCAUGCGUAAAC
CCGCCUUUCUAAGCGGAGAACAGAAGAAAGCAAUAGUAGAUCUGUUAUUCAAGACCAACCGCAAAGUGACAGUUAAGCAAUU
GAAAGAGGACUACUUUAAGAAAAUUGAAUGCUUCGAUUCUGUCGAGAUCUCCGGGGUAGAAGAUCGAUUUAAUGCGUCACU
UGGUACGUAUCAUGACCUCCUAAAGAUAAUUAAAGAUAAGGACUUCCUGGAUAACGAAGAGAAUGAAGAUAUCUUAGAAGAU
AUAGUGUUGACUCUUACCCUCUUUGAAGAUCGGGAAAUGAUUGAGGAAAGACUAAAAACAUACGCUCACCUGUUCGACGAU
AAGGUUAUGAAACAGUUAAAGAGGCGUCGCUAUACGGGCUGGGGAGCCUUGUCGCGGAAACUUAUCAACGGGAUAAGAGAC
AAGCAAAGUGGUAAAACUAUUCUCGAUUUUCUAAAGAGCGACGGCUUCGCCAAUAGGAACUUUAUGGCCCUGAUCCAUGAU
GACUCUUUAACCUUCAAAGAGGAUAUACAAAAGGCACAGGUUUCCGGACAAGGGGACUCAUUGCACGAACAUAUUGCGAAU
CUUGCUGGUUCGCCAGCCAUCAAAAAGGGCAUACUCCAGACAGUCAAAGUAGUGGAUGAGCUAGUUAAGGUCAUGGGACGU
CACAAACCGGAAAACAUUGUAAUCGAGAUGGCACGCGAAAAUCAAACGACUCAGAAGGGGCAAAAAAACAGUCGAGAGCGGA
UGAAGAGAAUAGAAGAGGGUAUUAAAGAACUGGGCAGCCAGAUCUUAAAGGAGCAUCCUGUGGAAAAUACCCAAUUGCAGAA
CGAGAAACUUUACCUCUAUUACCUACAAAAUGGAAGGGACAUGUAUGUUGAUCAGGAACUGGACAUAAACCGUUUAUCUGAU
UACGACGUCGAUCACAUUGUACCCCAAUCCUUUUUGAAGGACGAUUCAAUCGACAAUAAAGUGCUUACACGCUCGGAUAAGA
ACCGAGGGAAAAGUGACAAUGUUCCAAGCGAGGAAGUCGUAAAGAAAAUGAAGAACUAUUGGCGGCAGCUCCUAAAUGCGA
AACUGAUAACGCAAAGAAAGUUCGAUAACUUAACUAAAGCUGAGAGGGGUGGCUUGUCUGAACUUGACAAGGCCGGAUUUA
UUAAACGUCAGCUCGUGGAAACCCGCGCCAUCACAAAGCAUGUUGCGCAGAUACUAGAUUCCCGAAUGAAUACGAAAUACGA
CGAGAACGAUAAGCUGAUUCGGGAAGUCAAAGUAAUCACUUUAAAGUCAAAAUUGGUGUCGGACUUCAGAAAGGAUUUUCAA
UUCUAUAAAGUUAGGGAGAUAAAUAACUACCACCAUGCGCACGACGCUUAUCUUAAUGCCGUCGUAGGGACCGCACUCAUU
AAGAAAUACCCGAAGCUAGAAAGUGAGUUUGUGUAUGGUGAUUACAAAGUUUAUGACGUCCGUAAGAUGAUCGCGAAAAGC
GAACAGGAGAUAGGCAAGGCUACAGCCAAAUACUUCUUUUAUUCUAACAUUAUGAAUUUCUUUAAGACGGAAAUCACUCUGG
CAAACGGAGAGAUACGCAAACGACCUUUAAUUGAAACCAAUGGGGAGACAGGUGAAAUCGUAUGGGAUAAGGGCCGGGACU
UCGCGACGGUGAGAAAAGUUUUGUCCAUGCCCCAAGUCAACAUAGUAAAGAAAACUGAGGUGCAGACCGGAGGGUUUUCAA
AGGAAUCGAUUCUUCCAAAAAGGAAUAGUGAUAAGCUCAUCGCUCGUAAAAAGGACUGGGACCCGAAAAAGUACGGUGGCU
UCGAUAGCCCUACAGUUGCCUAUUCUGUCCUAGUAGUGGCAAAAGUUGAGAAGGGAAAAUCCAAGAAACUGAAGUCAGUCA
AAGAAUUAUUGGGGAUAACGAUUAUGGAGCGCUCGUCUUUUGAAAAGAACCCCAUCGACUUCCUUGAGGCGAAAGGUUACA
AGGAAGUAAAAAAGGAUCUCAUAAUUAAACUACCAAAGUAUAGUCUGUUUGAGUUAGAAAAUGGCCGAAAACGGAUGUUGGC
UAGCGCCGGAGAGCUUCAAAAGGGGAACGAACUCGCACUACCGUCUAAAUACGUGAAUUUCCUGUAUUUAGCGUCCCAUUA
CGAGAAGUUGAAAGGUUCACCUGAAGAUAACGAACAGAAGCAACUUUUUGUUGAGCAGCACAAACAUUAUCUCGACGAAAUC
AUAGAGCAAAUUUCGGAAUUCAGUAAGAGAGUCAUCCUAGCUGAUGCCAAUCUGGACAAAGUAUUAAGCGCAUACAACAAGC
ACAGGGAUAAACCCAUACGUGAGCAGGCGGAAAAUAUUAUCCAUUUGUUUACUCUUACCAACCUCGGCGCUCCAGCCGCAU
UCAAGUAUUUUGACACAACGAUAGAUCGCAAACGAUACACUUCUACCAAGGAGGUGCUAGACGCGACACUGAUUCACCAAUC
CAUCACGGGAUUAUAUGAAACUCGGAUAGAUUUGUCACAGCUUGGGGGUGACGGAUCCCCCAAGAAGAAGAGGAAAGUCUC
GAGCGACUACAAAGACCAUGACGGUGAUUAUAAAGAUCAUGACAUCGAUUACAAGGAUGACGAUGACAAGGCUGCAGGAUG
ACCGGUCAUCAUCACCAUCACCAUUGAGU

eGFP RNA sequence (GenBank: MH450170.1)

GGGAGACCCUCGAGGACAGAUCGCCUGGAGACGCCAUCCACGCUGUUUUGACCUCCAUAGAAGACACCGGGACCGAUCCA
GCCUCCGCGGCCGGGAACGGUGCAUUGGAACGCGGAUUCCCCGUGCCAAGAGUGACUCACCGUCCUUGACACGAUGGUGA

GCAAGGGCGAGGAGCUGUUCACCGGGGUGGUGCCCAUCCUGGUCGAGCUGGACGGCGACGUAAACGGCCACAAGUUCAGC
GUGUCCGGCGAGGGCGAGGGCGAUGCCACCUACGGCAAGCUGACCCUGAAGUUCAUCUGCACCACCGGCAAGCUGCCCGU
GCCCUGGCCCACCCUCGUGACCACCCUGACCUACGGCGUGCAGUGCUUCAGCCGCUACCCCGACCACAUGAAGCAGCACG
ACUUCUUCAAGUCCGCCAUGCCCGAAGGCUACGUCCAGGAGCGCACCAUCUUCUUCAAGGACGACGGCAACUACAAGACCC
GCGCCGAGGUGAAGUUCGAGGGCGACACCCUGGUGAACCGCAUCGAGCUGAAGGGCAUCGACUUCAAGGAGGACGGCAAC
AUCCUGGGGCACAAGCUGGAGUACAACUACAACAGCCACAACGUCUAUAUCAUGGCCGACAAGCAGAAGAACGGCAUCAAG
GUGAACUUCAAGAUCCGCCACAACAUCGAGGACGGCAGCGUGCAGCUCGCCGACCACUACCAGCAGAACACCCCCAUCGGC
GACGGCCCCGUGCUGCUGCCCGACAACCACUACCUGAGCACCCAGUCCGCCCUGAGCAAAGACCCCAACGAGAAGCGCGAU
CACAUGGUCCUGCUGGAGUUCGUGACCGCCGCCGGGAUCACUCUCGGCAUGGACGAGCUGUACAAGUAACGGGUGGCAUC
CCUGUGACCCCUCCCCAGUGCCUCUCCUGGCCCUGGAAGUUGCCACUCCAGUGCCCACCAGCCUUGUCCUAAUAAAAUUAA
GUUGCAUCAAGCU

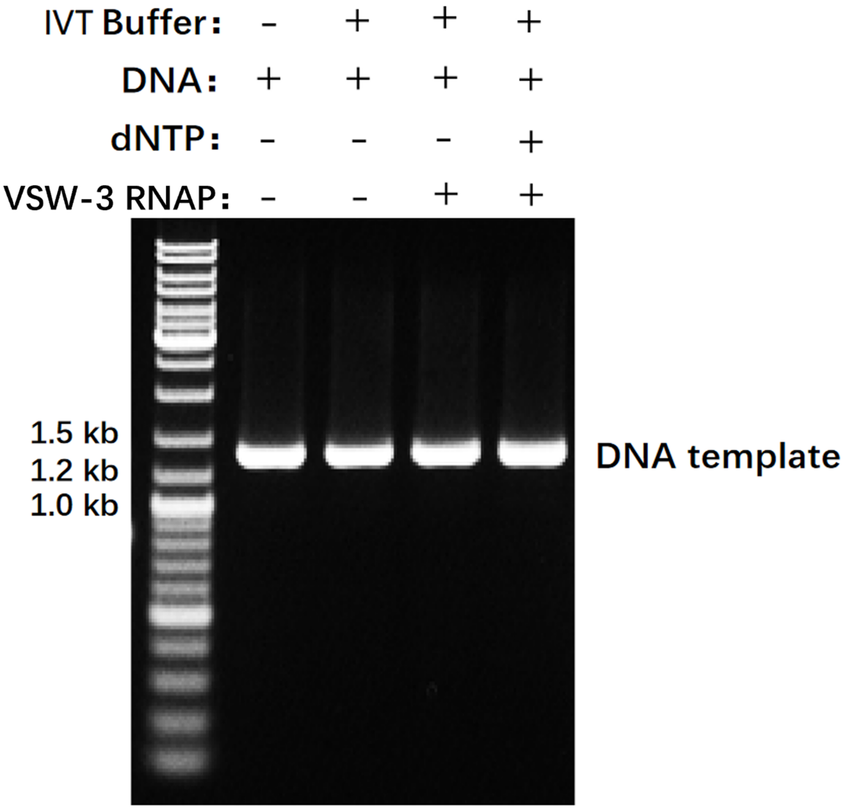

Figure S1. In the IVT reaction (40 mM Tris-HCl pH 8.0, 16 mM $MgCl_2$, 5 mM DTT, 2 mM spermidine, 50 ng/μl copGFP DNA template), DNase or DNA polymerase activity was not detected from purified VSW-3 RNAP at high concentration (2 μM).

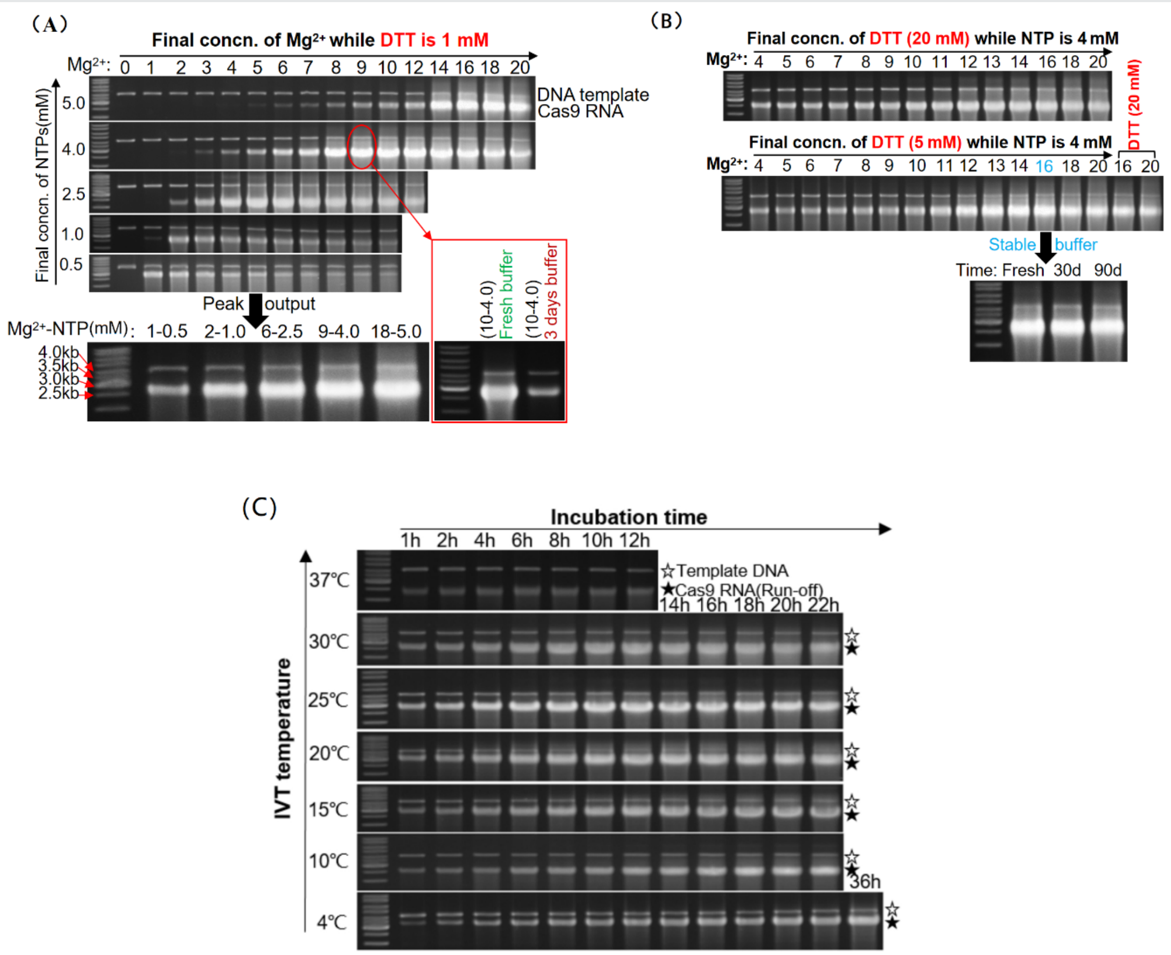

Figure S2. Screening for VSW-3 RNAP IVT conditions. **(A)** The optimal Mg^{2+}-NTP concentration in the presence of 1 mM DTT. RNA yield with various optimal Mg^{2+}-NTP concentration combination was further compared (gel in the dotted box). The stability of the optimal VSW-3 RNAP IVT buffer with 1 mM DTT was examined (gel in the solid box). **(B)** Screening for the optimal DTT-Mg^{2+} concentration for the stable and high-yield VSW-3 RNAP IVT buffer. The stability of the high-yield VSW-3 RNAP IVT buffer containing 16 mM Mg^{2+}, 4 mM NTP and 5 mM DTT was examined (gel in the solid box). **(C)** Yield of cas9 RNA in VSW-3 RNAP IVT with various incubation temperature and time. The gel bands corresponding to DNA templates were indicated by empty stars and those corresponding to run-off cas9 RNA indicated by filled stars. Maximum yield was obtained at 25℃ for 12 hours. At lower temperatures, extended incubation increased the yield.

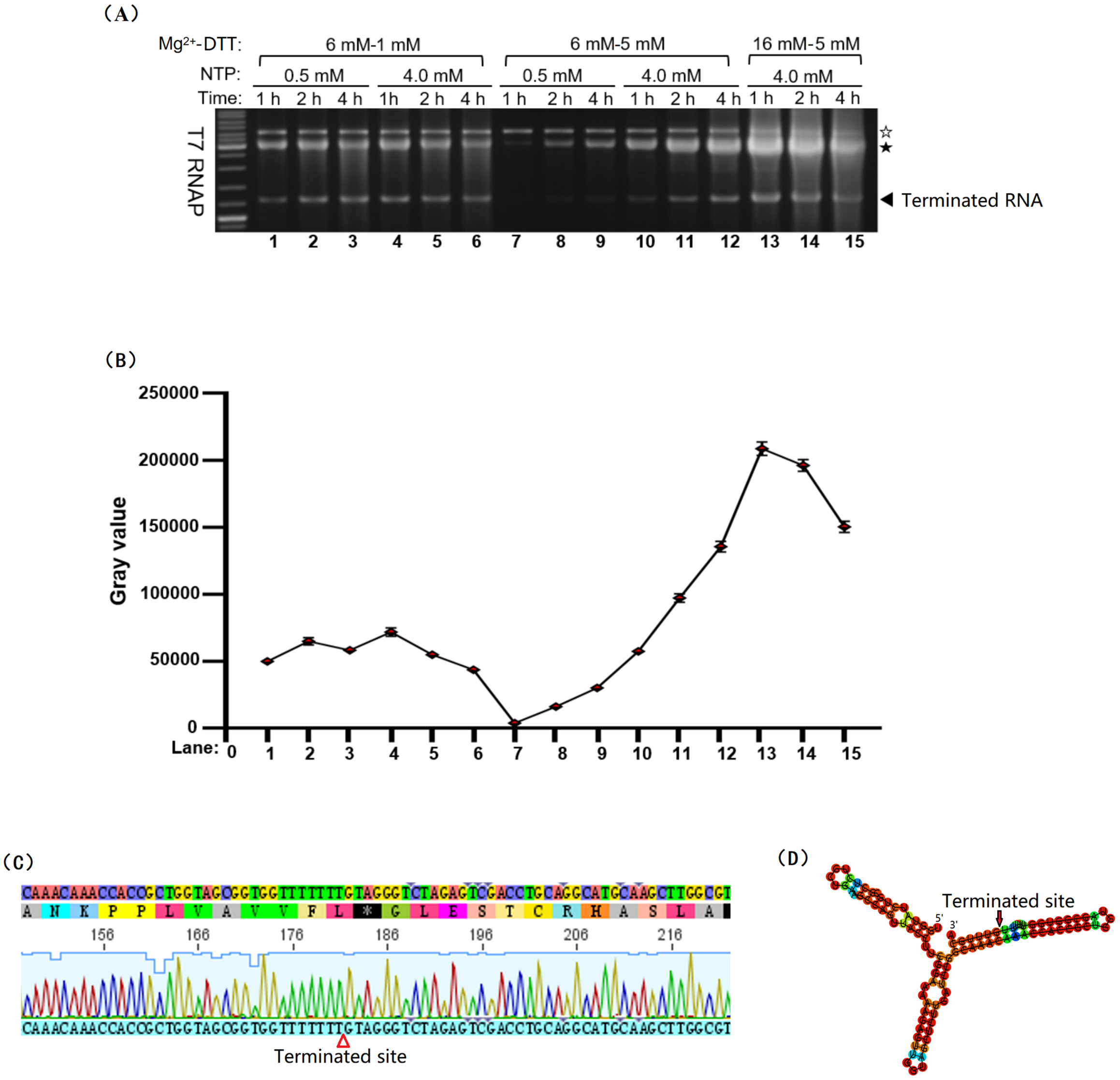

Figure S3. IVT yield of T7 RNAP with optimal IVT buffer for VSW-3 RNAP**. (A)** The optimized $MgCl_2$-NTP-DTT concentration (16-4-5 mM) for VSW-3 RNAP also applies to T7 RNAP. While extended incubation time (>1 hour) decreases the yield of run-off transcripts of T7 RNAP at 37℃. The gel bands corresponding to DNA templates were indicated by empty star and those corresponding to run-off cas9 RNA indicated by filled star. **(B)** Gray-scale quantitation of the run-off cas9 RNA in **(A)** by image J software revealed that T7 RNAP reaches its maximum yield in 1 hour with the optimized IVT conditions at 37℃. **(C)** 3' RACE of the terminated pUC19-RNA (indicated by an arrow in **(A)**) showed that the termination site of pUC19-RNA was located at the origin of replication of pUC19 plasmid. **(D)** RNAfold revealed that this region forms a stem-loop structure resembling a class I T7 Φ terminator.

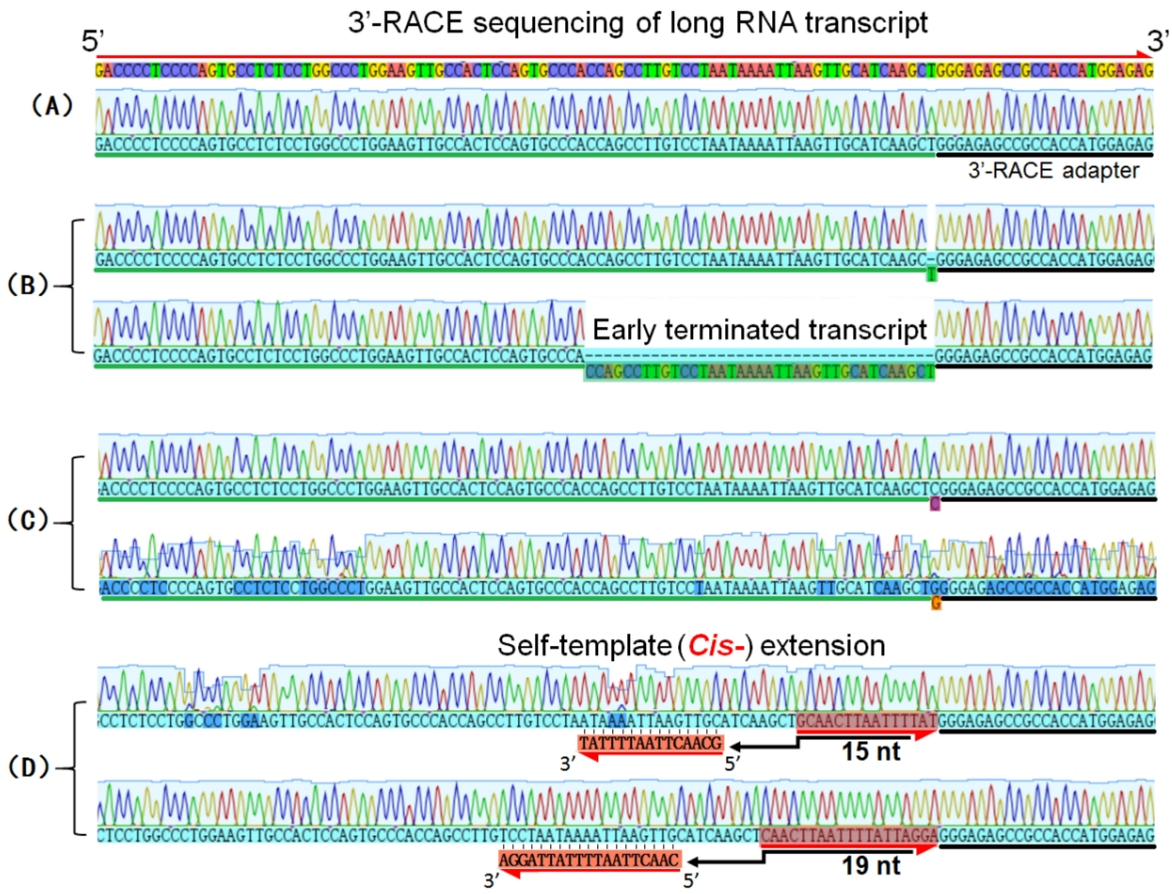

Figure S4. 3'-RACE detection of the self-templated (*Cis*-) 3' extension of eGFP RNA by VSW-3 and T7 RNAP. **(A)** Only one sequencing result from VSW-3 and T7 RNAP group showed no 3' extension respectively. **(B)** In VSW-3 RNAP group, three sequences showed 1 nt 3' truncation and one sequence showed larger 3' truncation. **(C)** In T7 RNAP group, a single nt "C" or "G" extension was observed. **(D)** In T7 RNAP group, two larger 3' extensions ("5-GCAACTTAATTTTAT-3 and 5-CAACTTAATTTTATTAGGA-3") were complementary to the sequences ("5-ATAAAATTAAGTTGC-3 and 5-TCCTAATAAAATTAAGTTG-3") near the 3' end of eGFP RNA.
